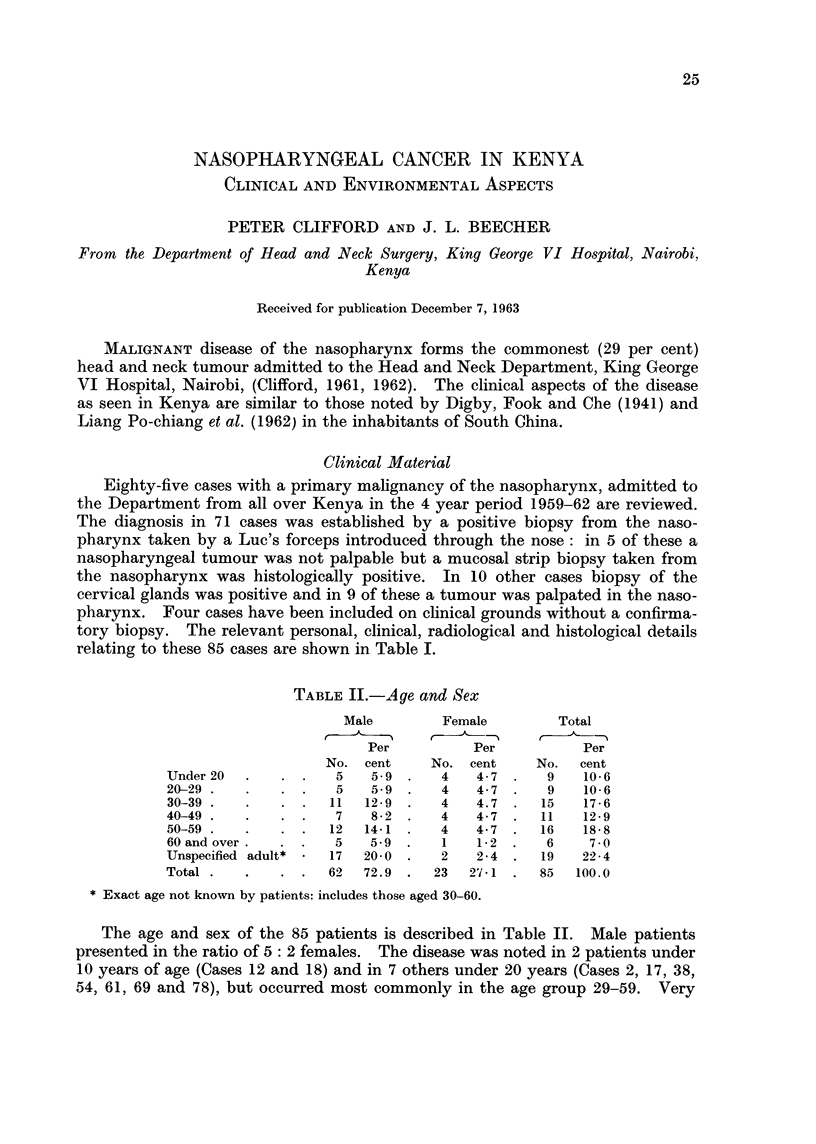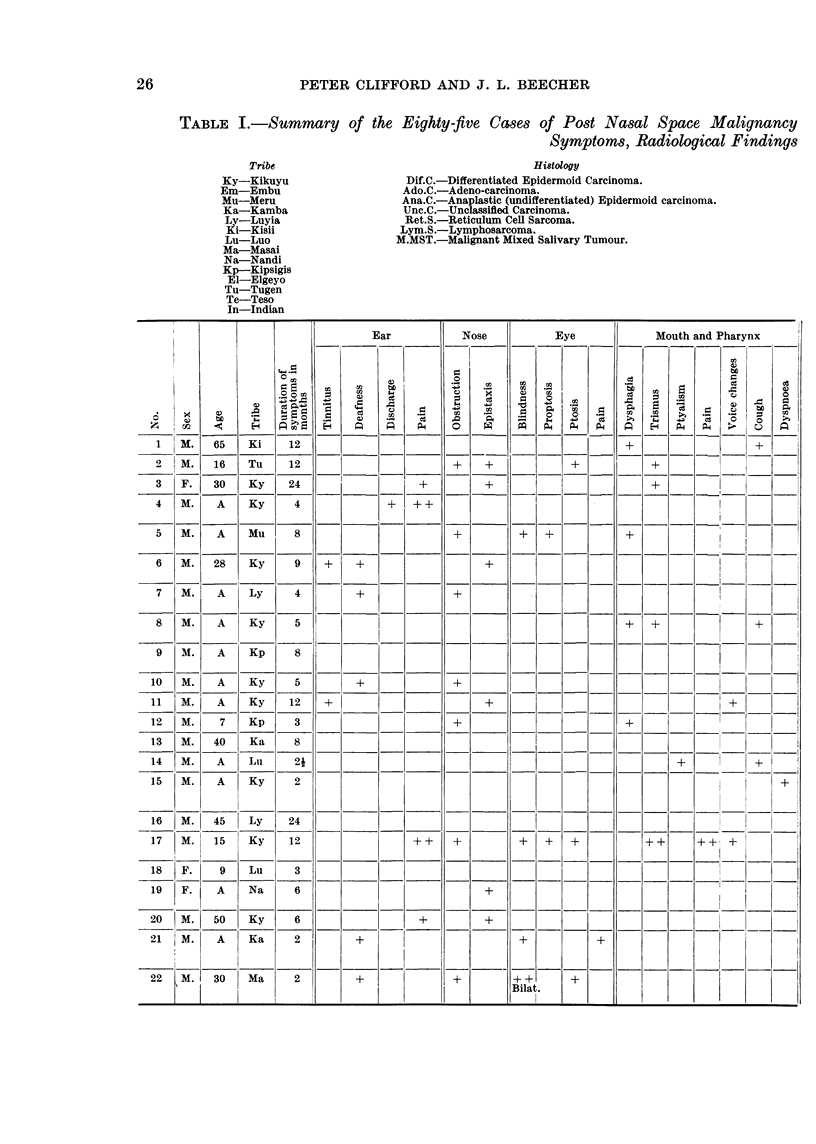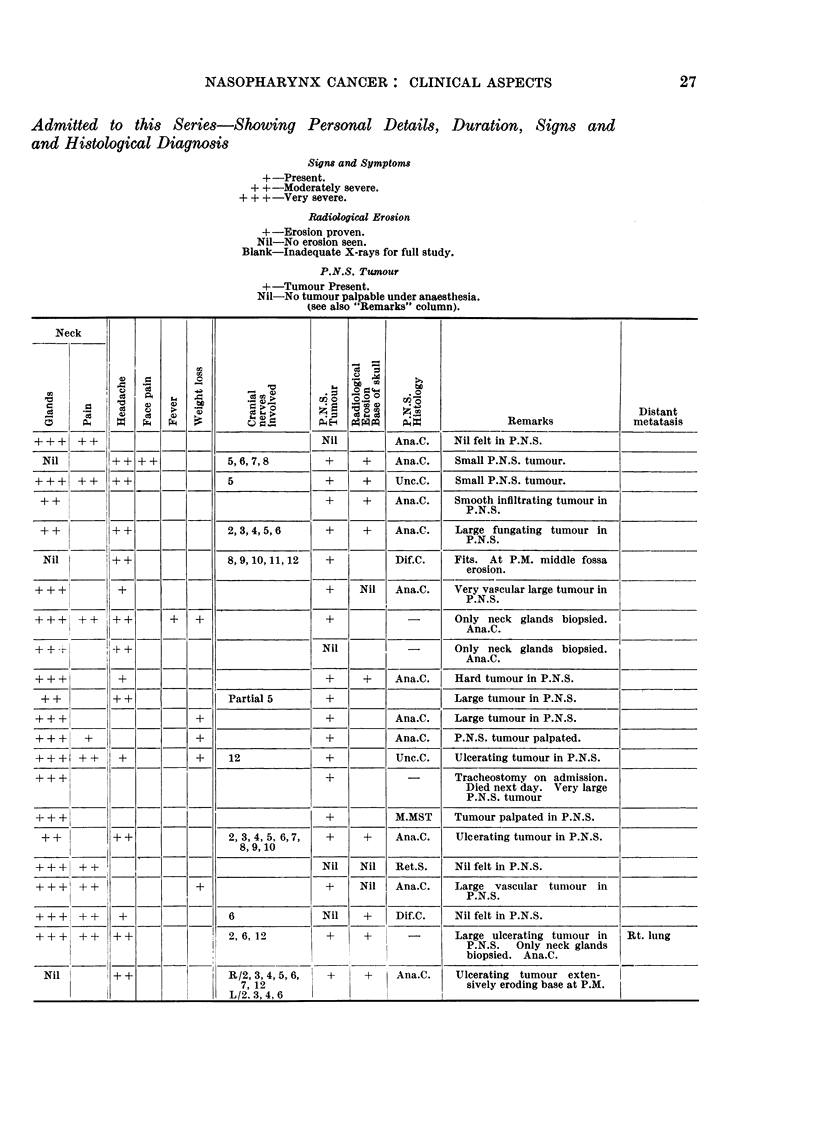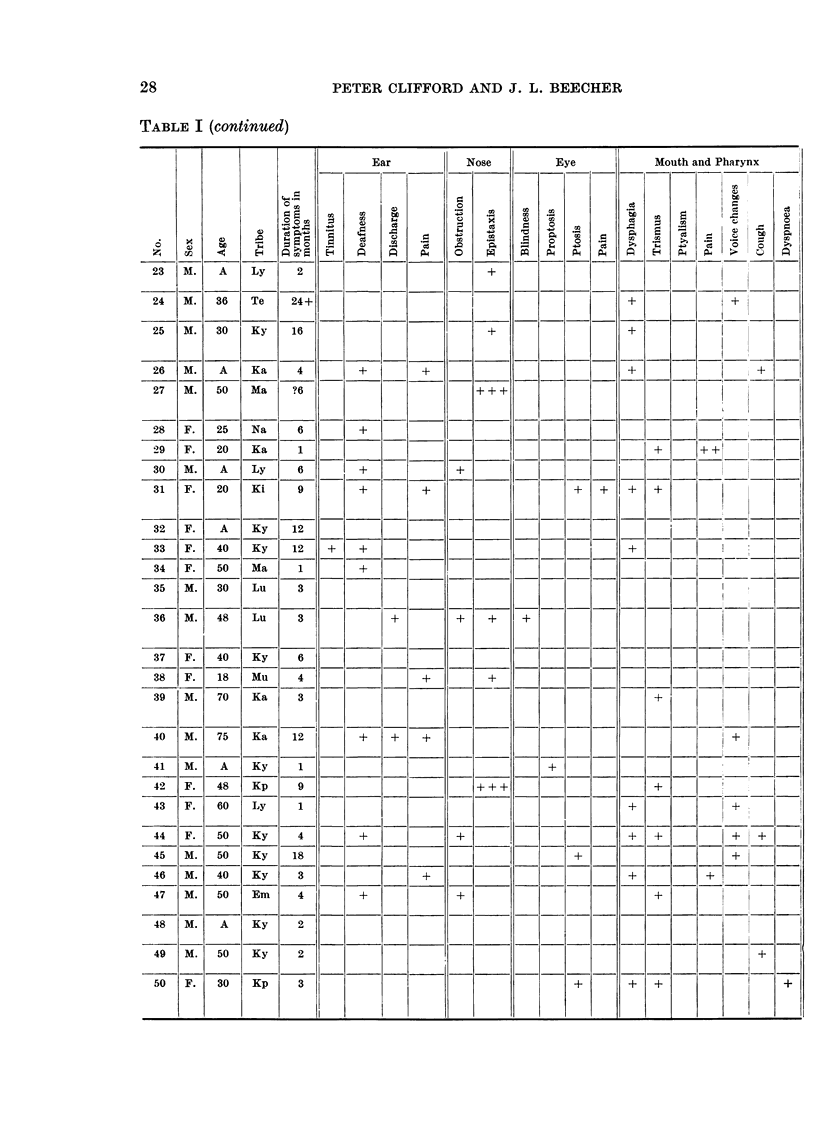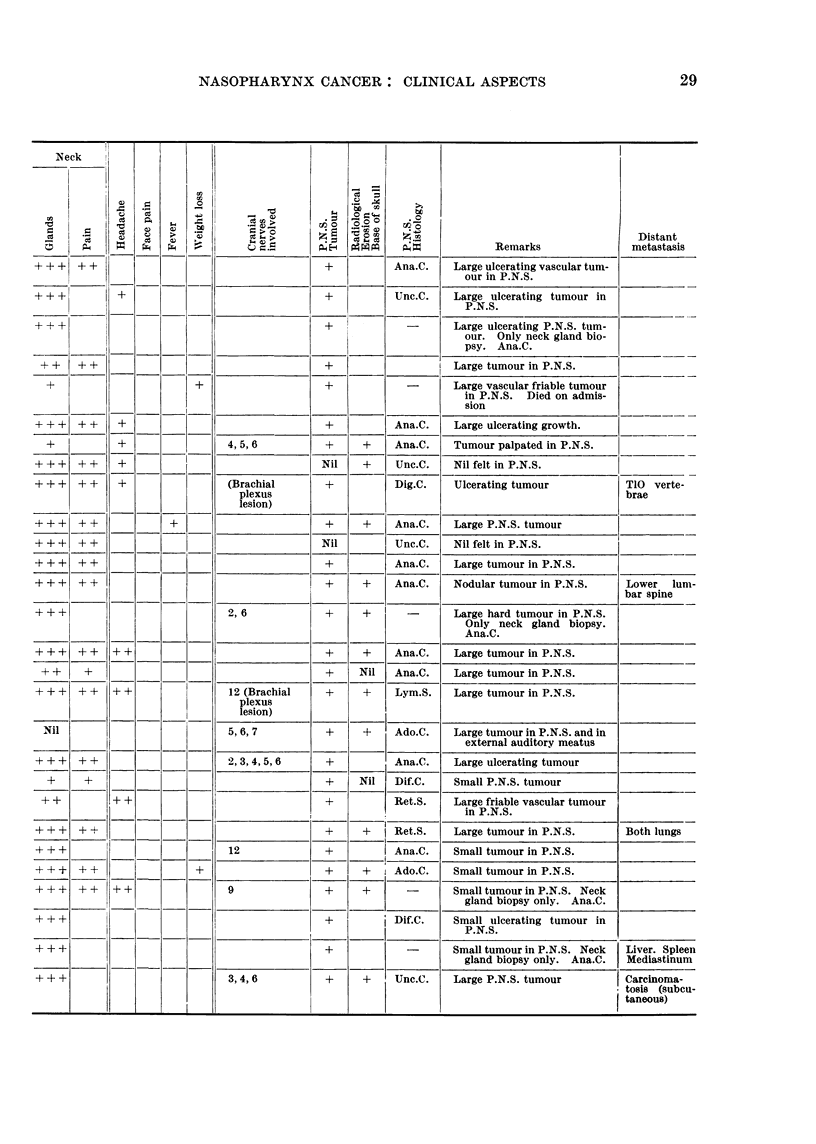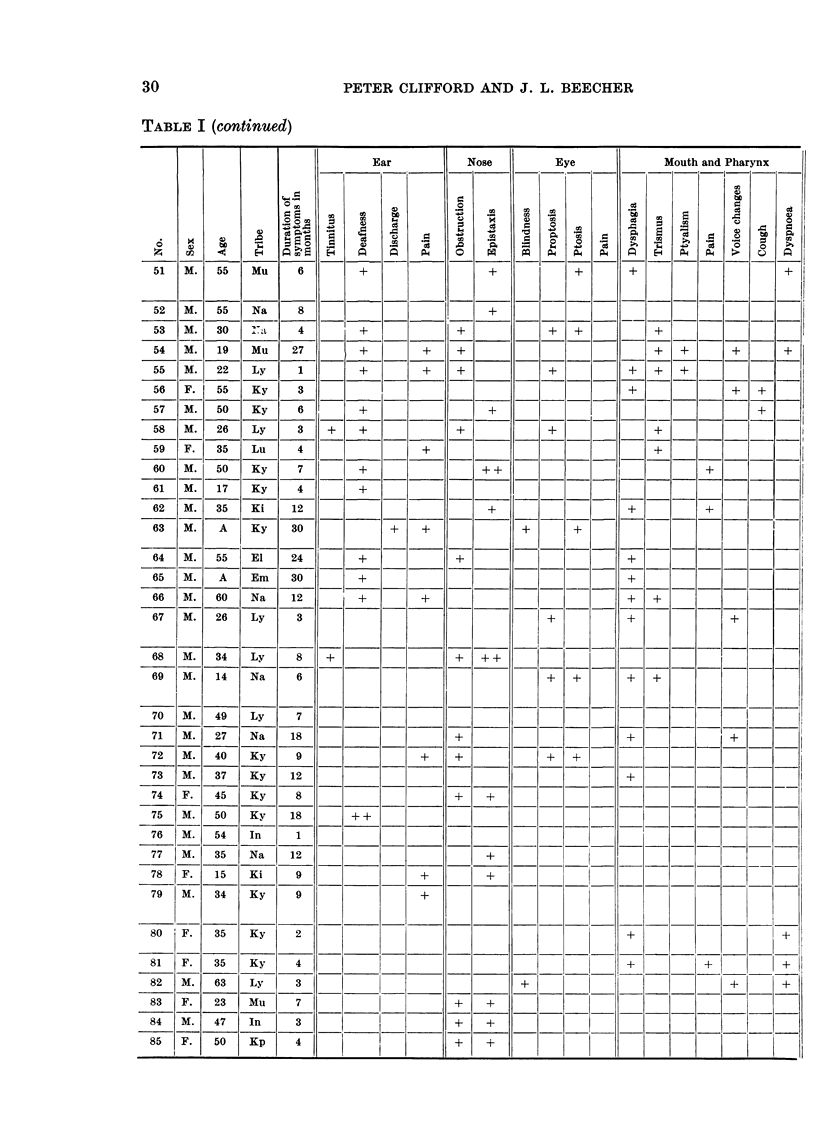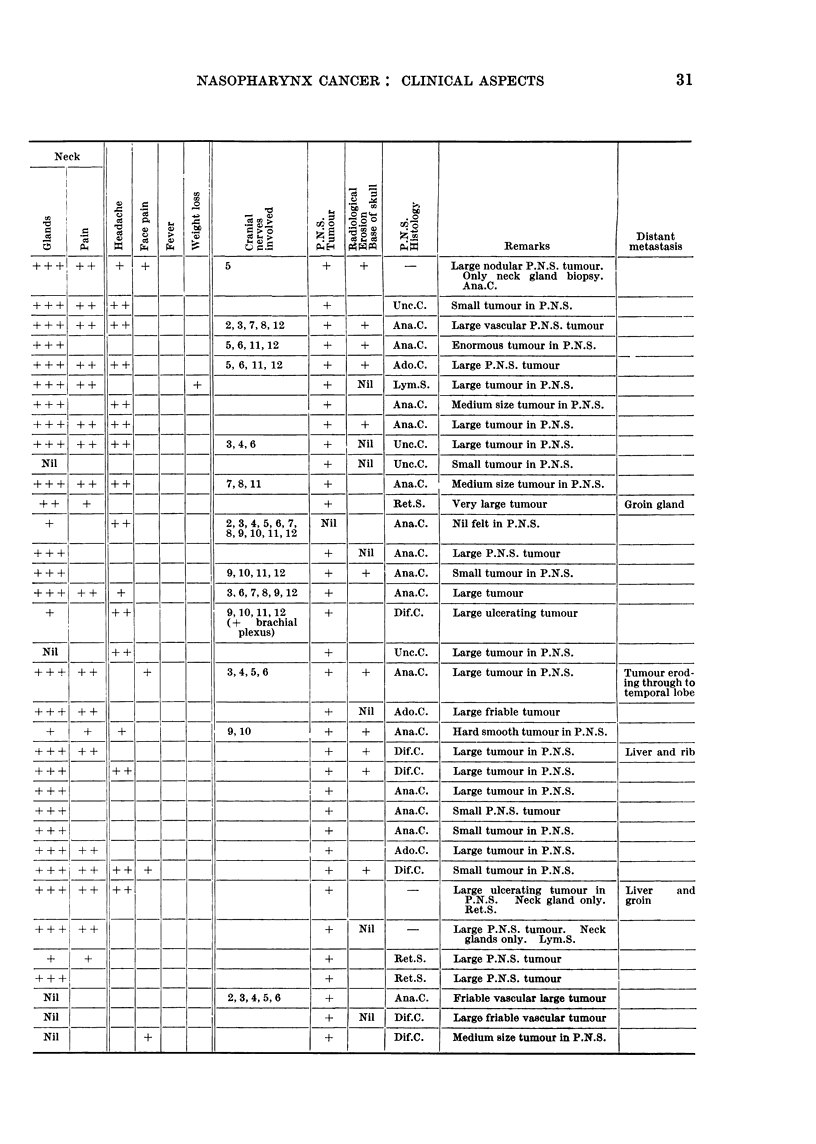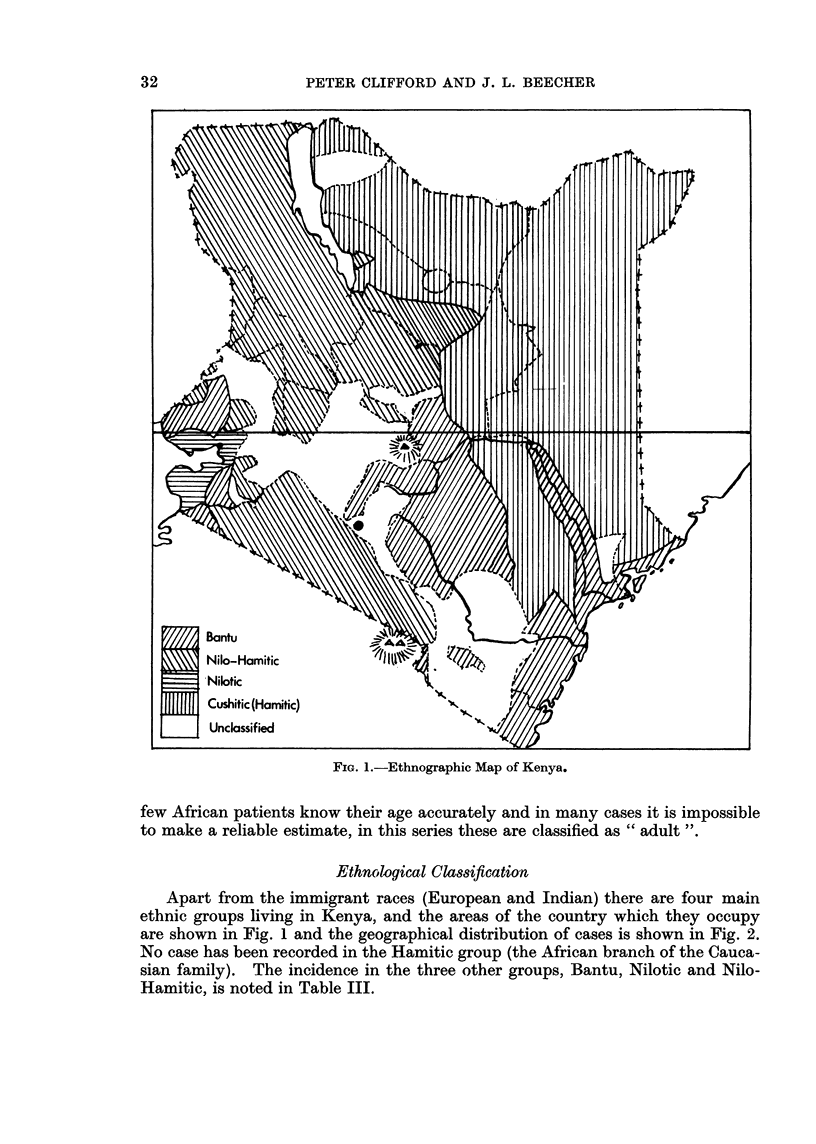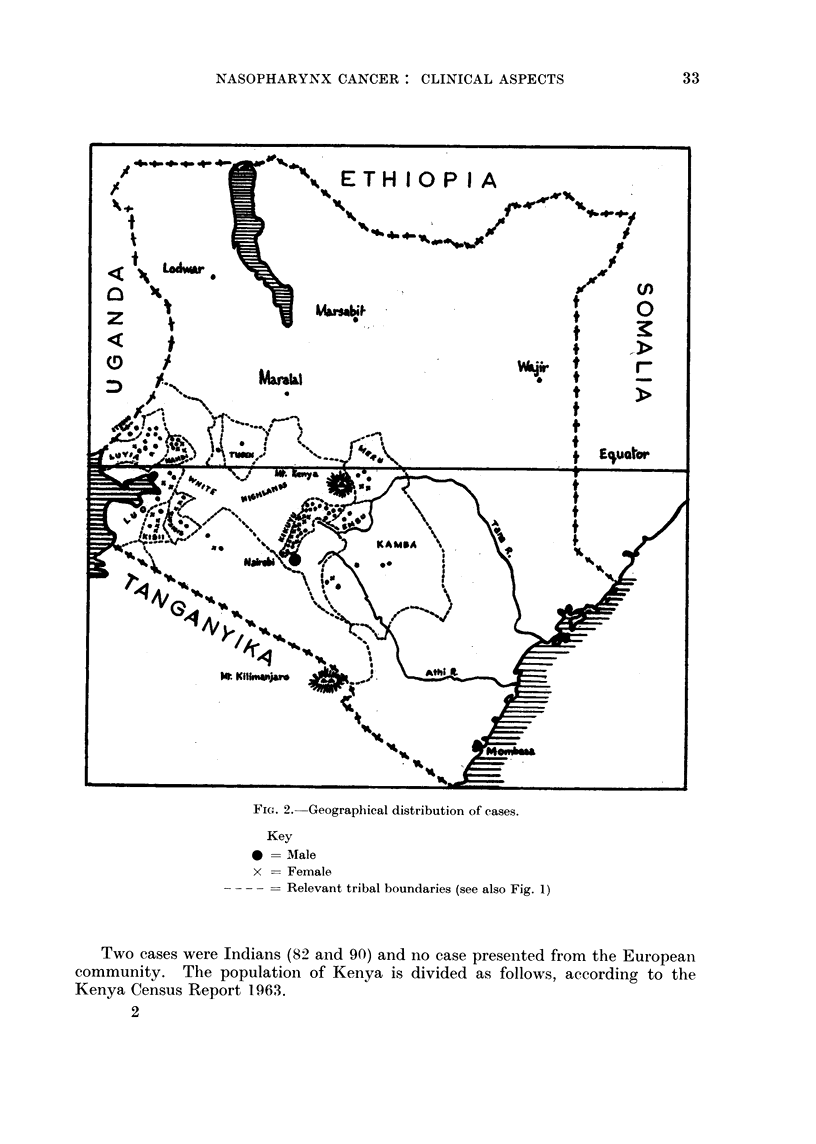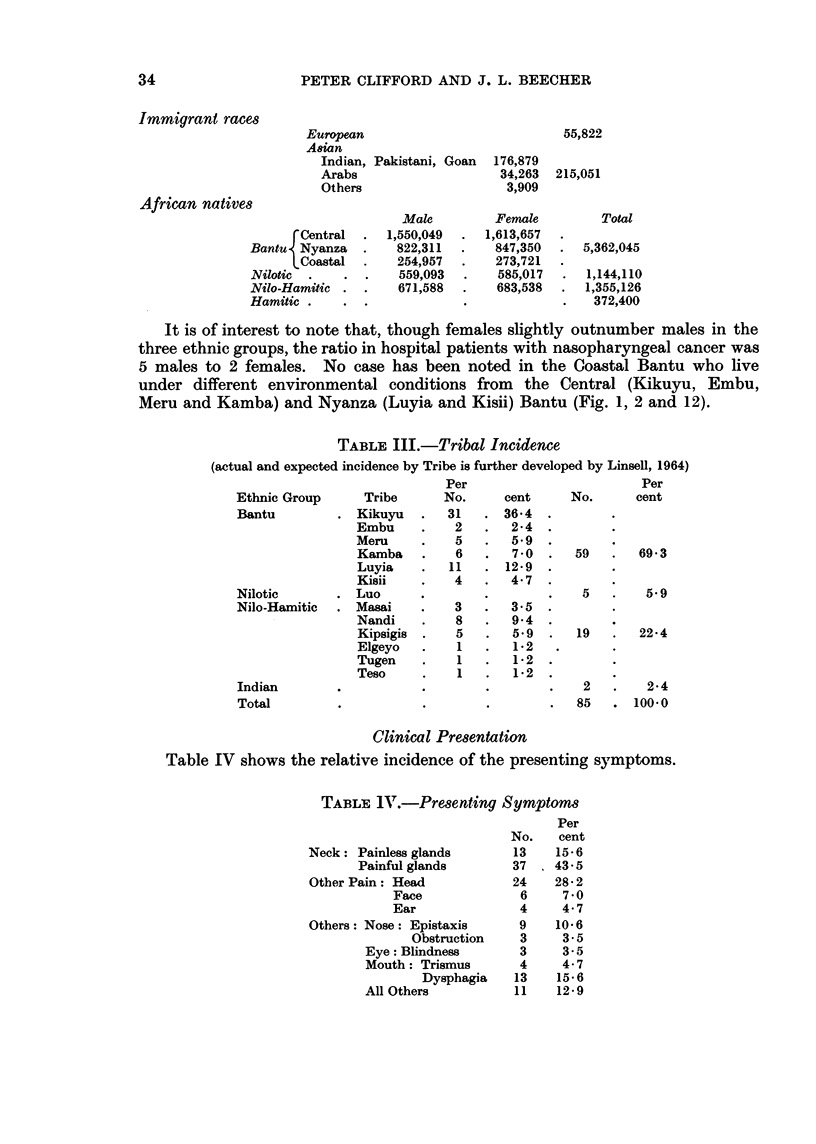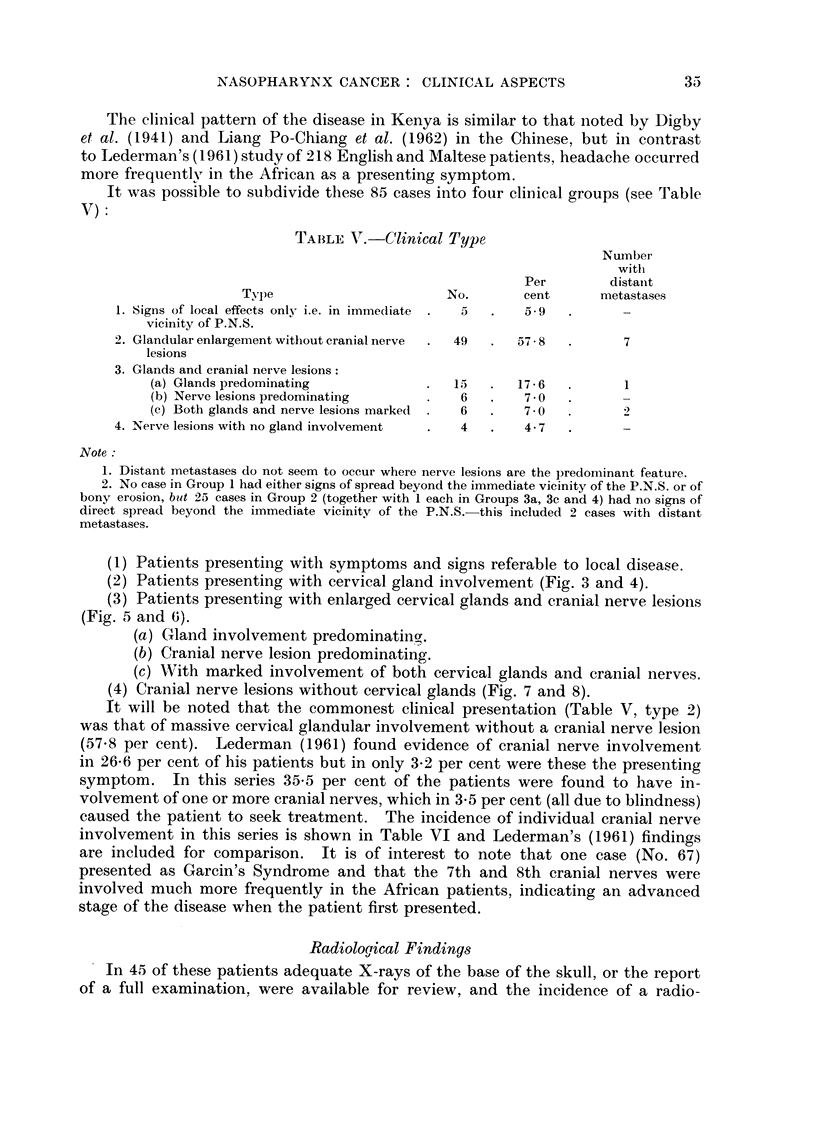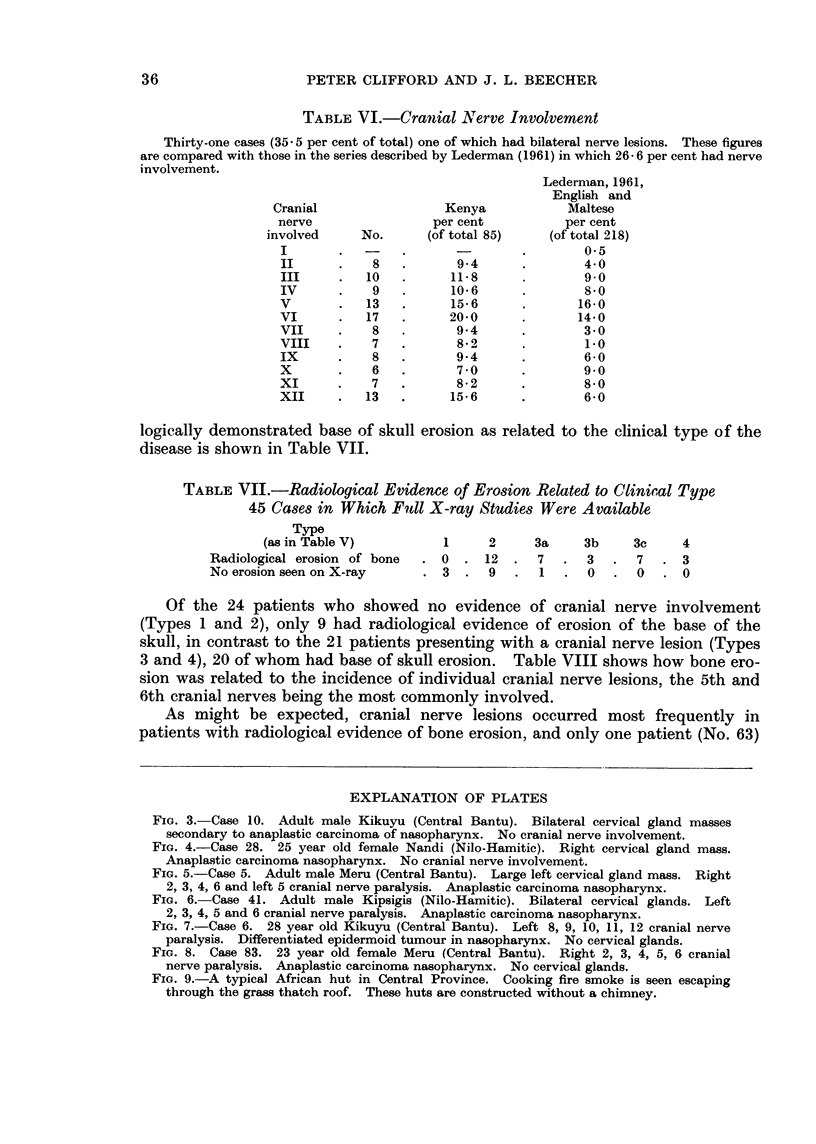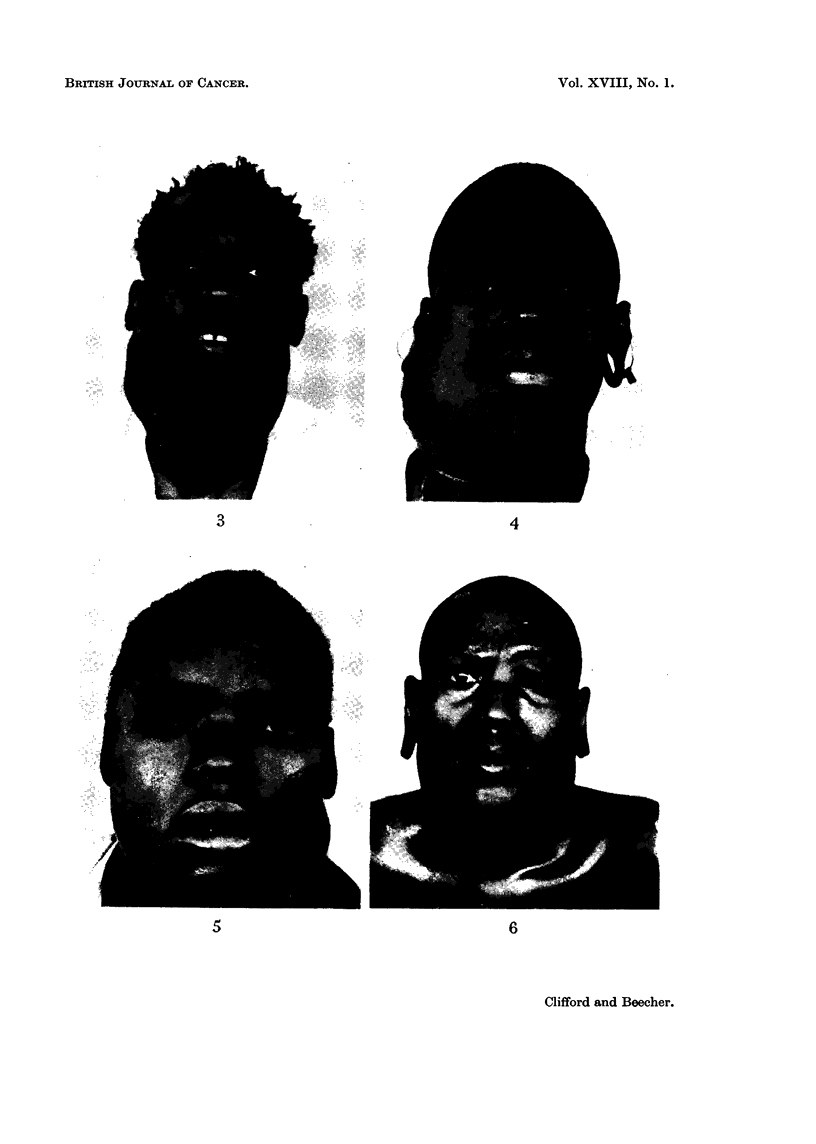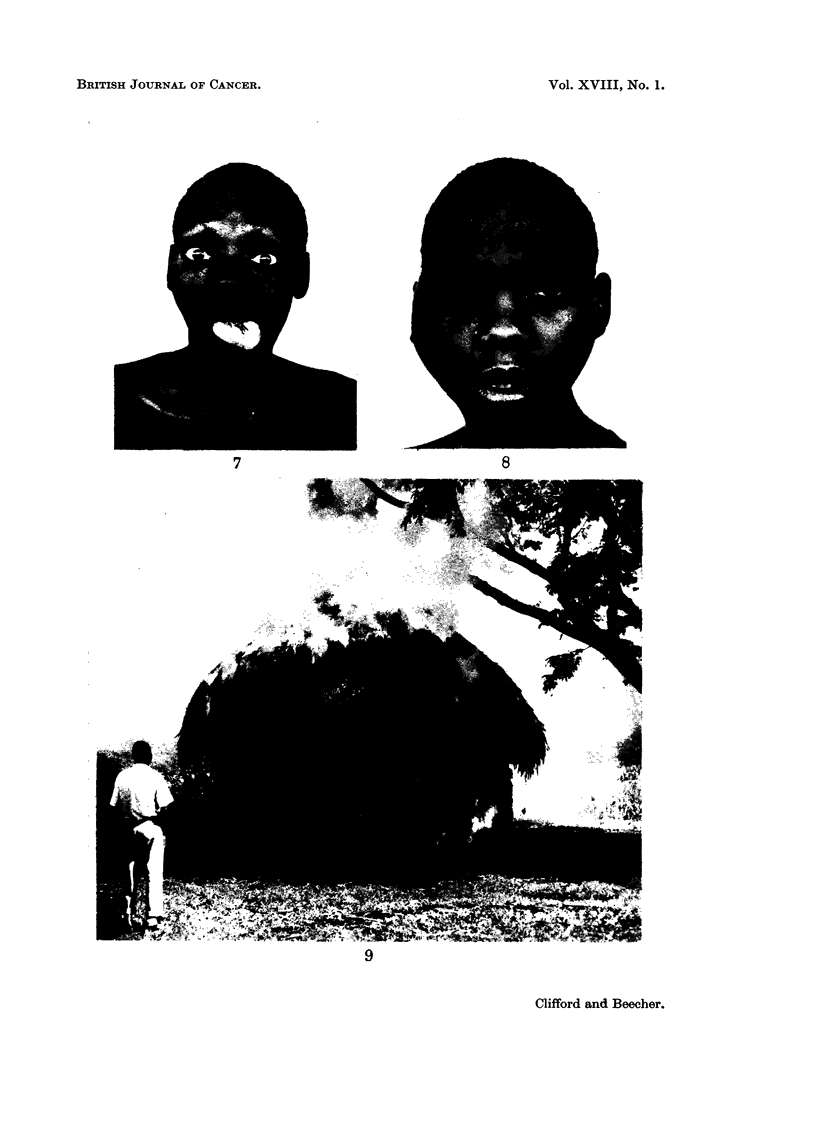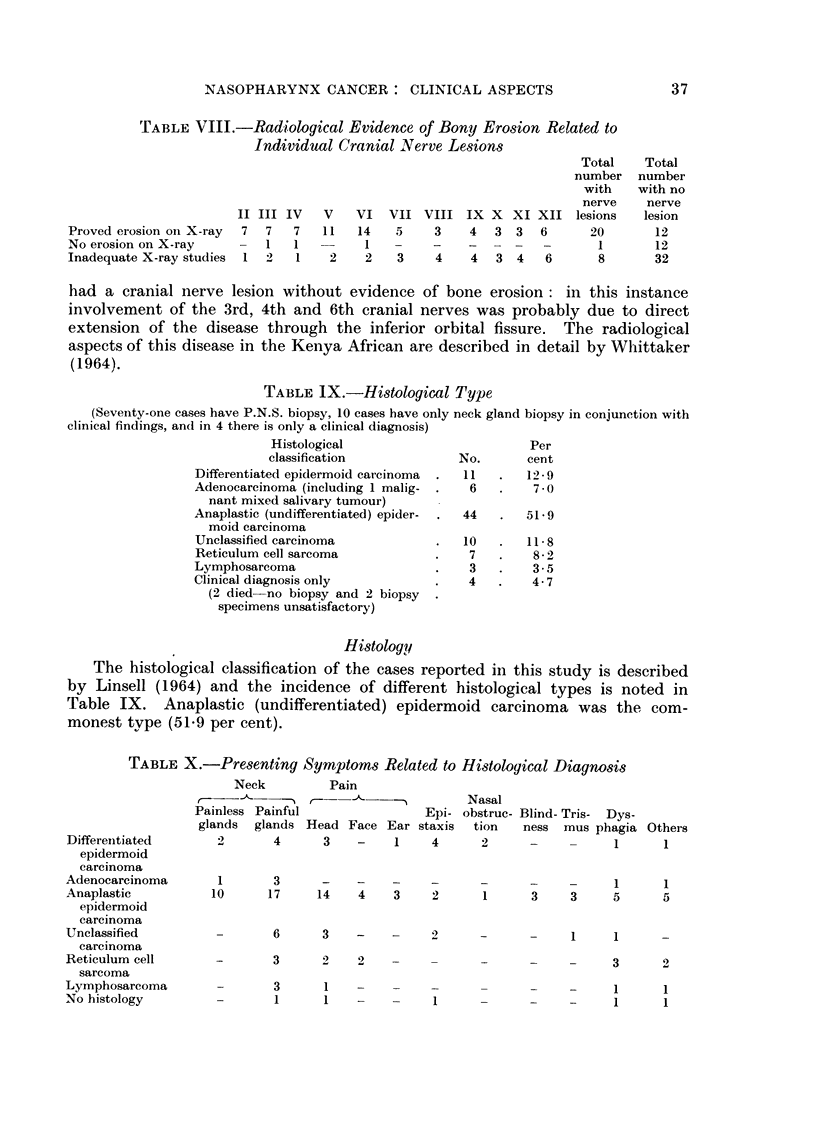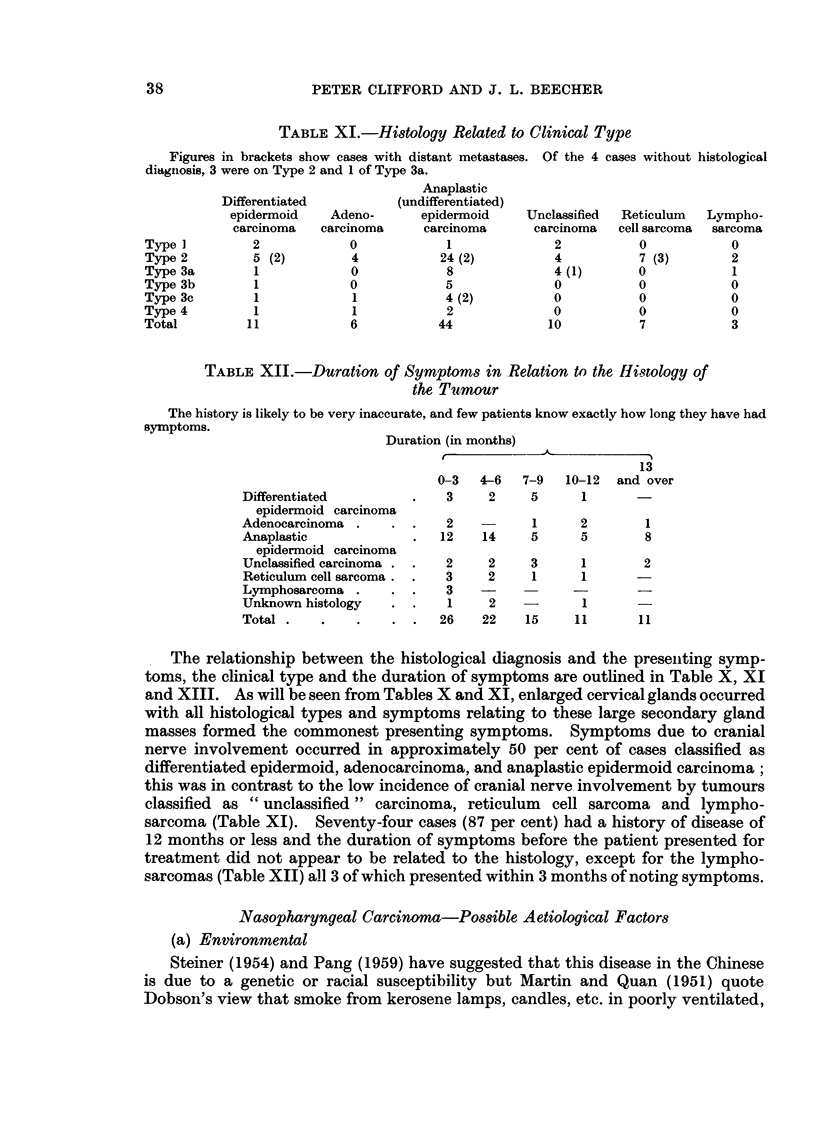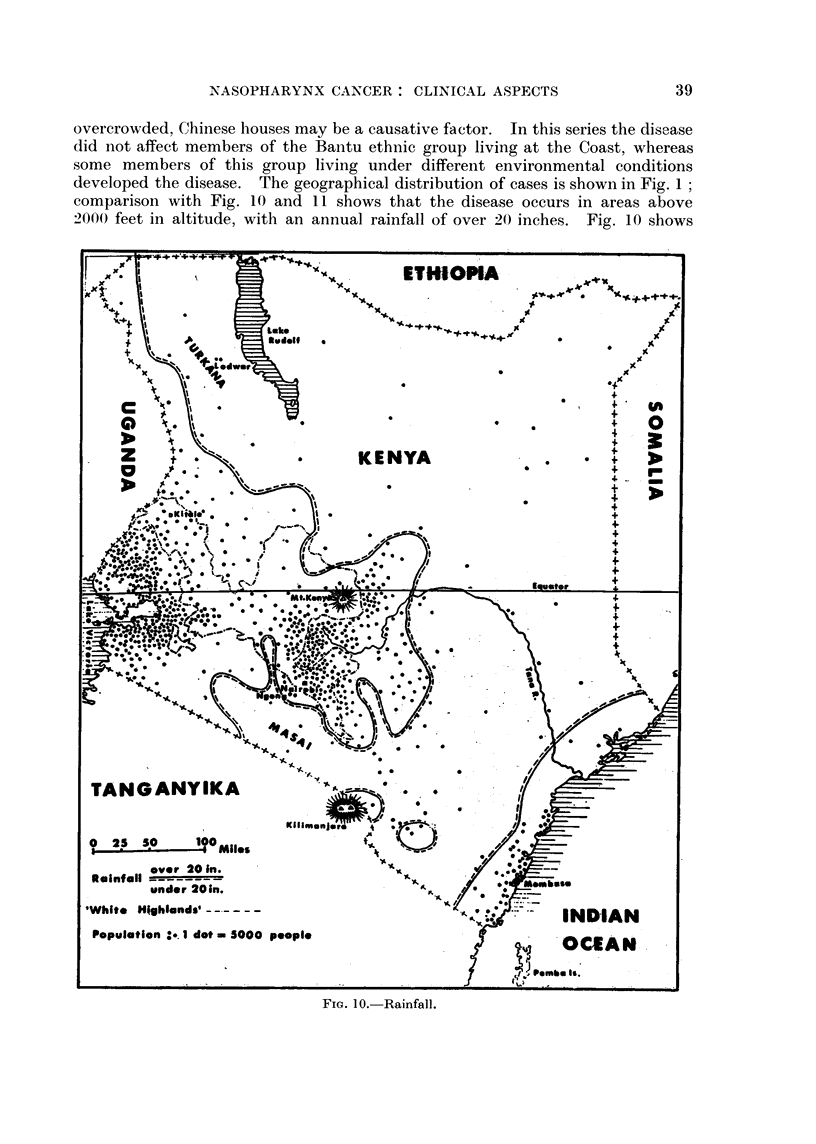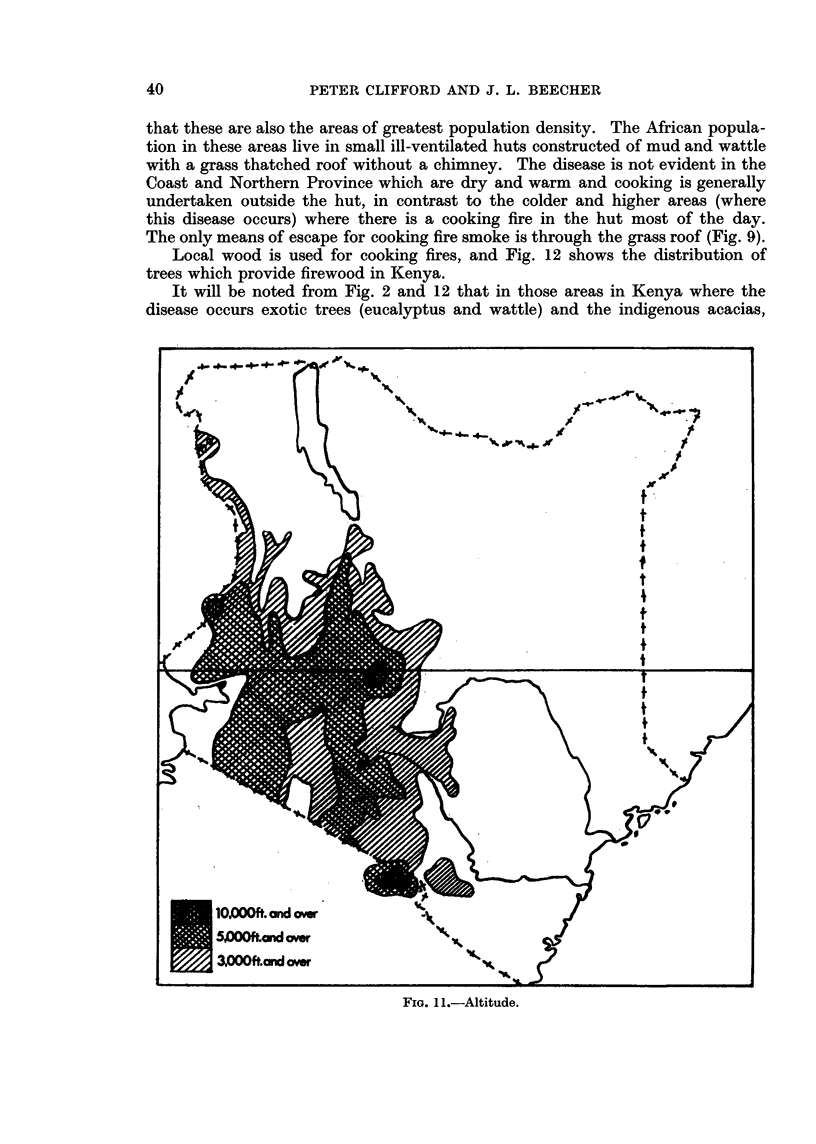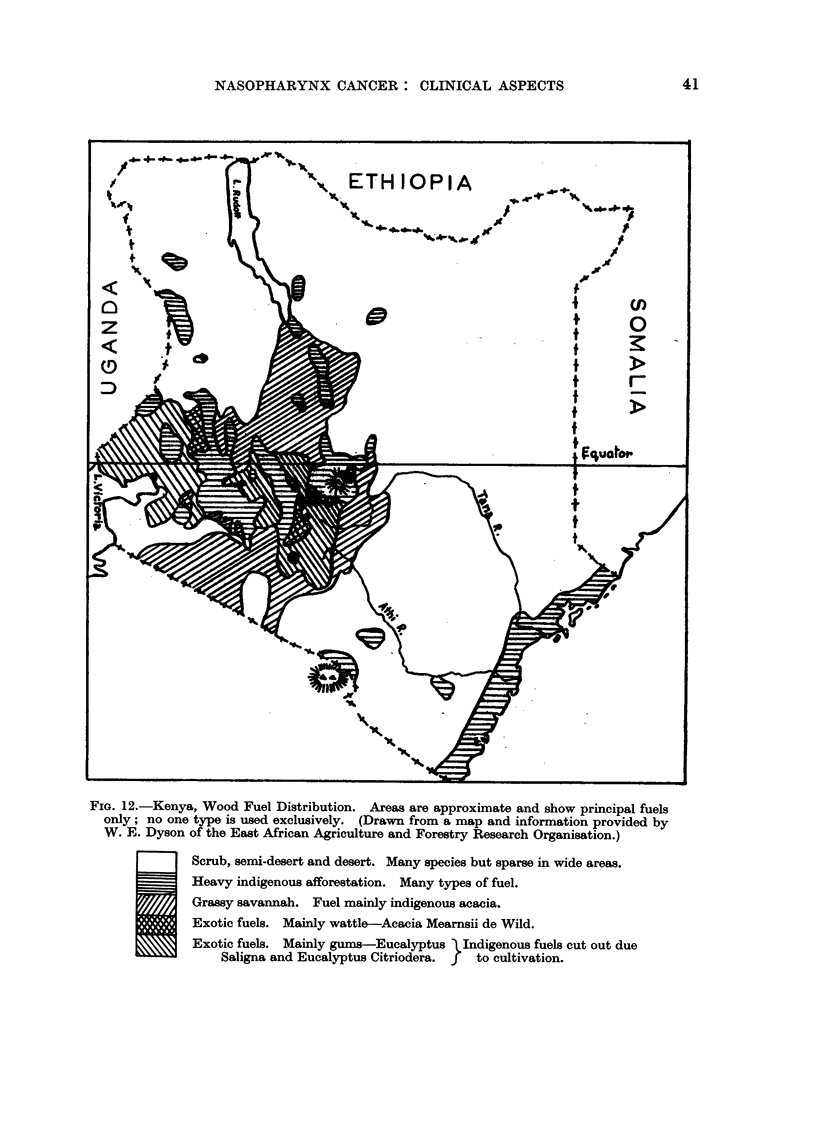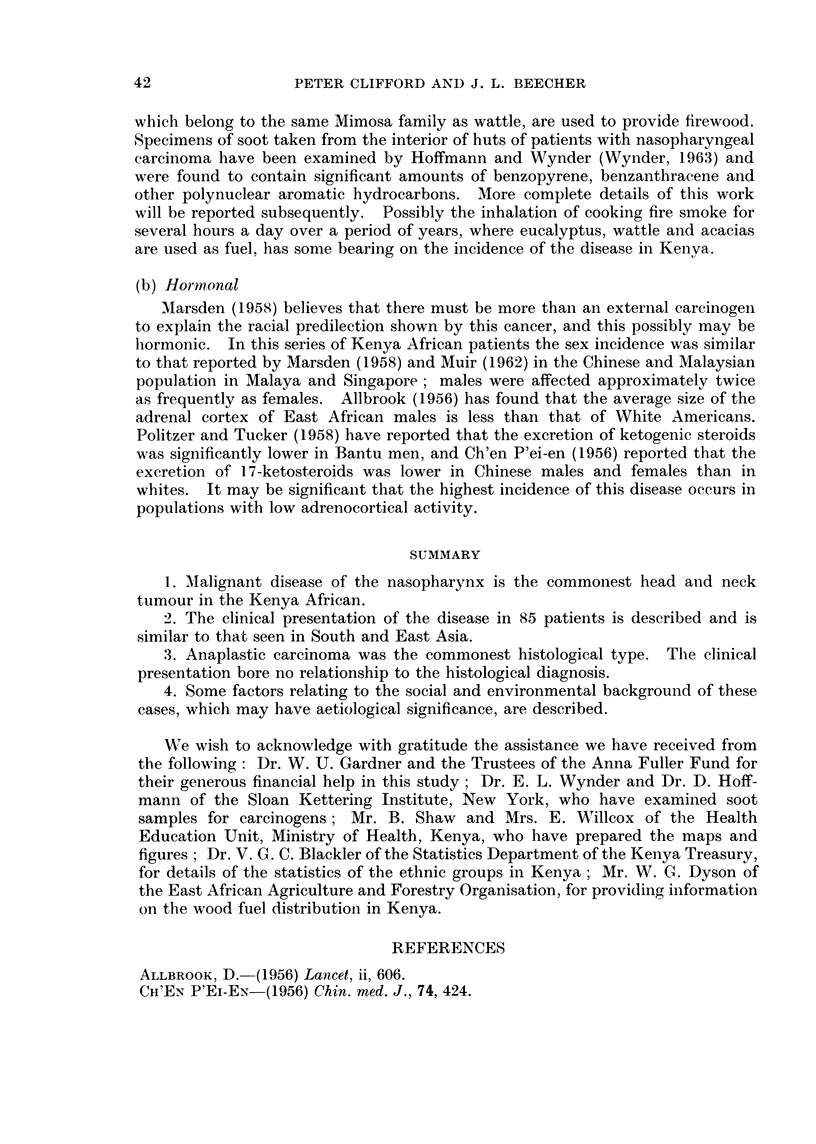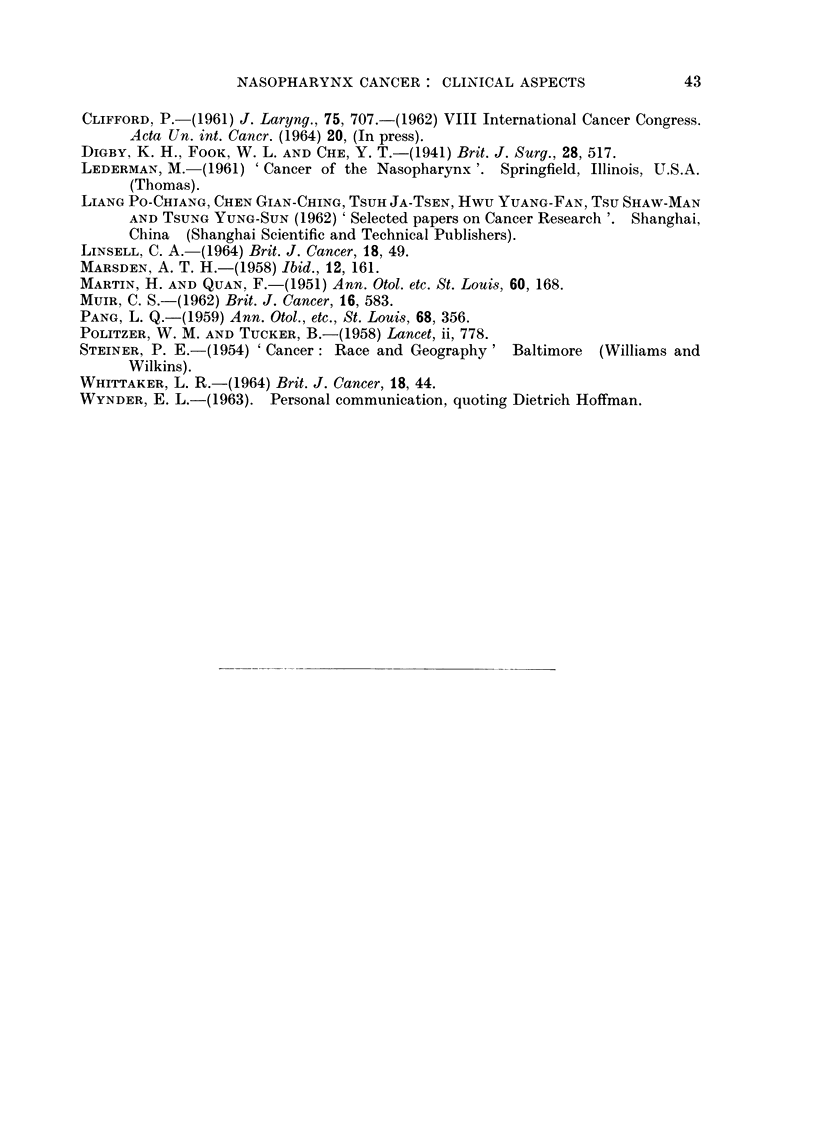# Nasopharyngeal Cancer in Kenya

**DOI:** 10.1038/bjc.1964.3

**Published:** 1964-03

**Authors:** Peter Clifford, J. L. Beecher

## Abstract

**Images:**


### NASOPHARYNGEAL CANCER IN KENYA

### Clinical and Environmental Aspects

PETER CLIFFORD AND J. L. BEECHER

*From the Department of Head and Neck Surgery, King George VI Hospital, Nairobi, Kenya*

Received for publication December 7, 1963

MALIGNANT disease of the nasopharynx forms the commonest (29 per cent) head and neck tumour admitted to the Head and Neck Department, King George VI Hospital, Nairobi, (Clifford, 1961, 1962). The clinical aspects of the disease as seen in Kenya are similar to those noted by Digby, Fook and Che (1941) and Liang Po-chiang *et al.* (1962) in the inhabitants of South China.

### *Clinical Material*

Eighty-five cases with a primary malignancy of the nasopharynx, admitted to the Department from all over Kenya in the 4 year period 1959–62 are reviewed. The diagnosis in 71 cases was established by a positive biopsy from the nasopharynx taken by a Luc's forceps introduced through the nose : in 5 of these a nasopharyngeal tumour was not palpable but a mucosal strip biopsy taken from the nasopharynx was histologically positive. In 10 other cases biopsy of the cervical glands was positive and in 9 of these a tumour was palpated in the nasopharynx. Four cases have been included on clinical grounds without a confirmatory biopsy. The relevant personal, clinical, radiological and histological details relating to these 85 cases are shown in Table I.

TABLE II.—*Age and Sex*

| | Male | | Female | | Total | |
|---|---|---|---|---|---|---|
| | No. | Per cent | No. | Per cent | No. | Per cent |
| Under 20 | 5 | 5·9 | 4 | 4·7 | 9 | 10·6 |
| 20–29 | 5 | 5·9 | 4 | 4·7 | 9 | 10·6 |
| 30–39 | 11 | 12·9 | 4 | 4.7 | 15 | 17·6 |
| 40–49 | 7 | 8·2 | 4 | 4·7 | 11 | 12·9 |
| 50–59 | 12 | 14·1 | 4 | 4·7 | 16 | 18·8 |
| 60 and over | 5 | 5·9 | 1 | 1·2 | 6 | 7·0 |
| Unspecified adult* | 17 | 20·0 | 2 | 2·4 | 19 | 22·4 |
| Total | 62 | 72.9 | 23 | 27·1 | 85 | 100.0 |

* Exact age not known by patients: includes those aged 30–60.

The age and sex of the 85 patients is described in Table II. Male patients presented in the ratio of 5 : 2 females. The disease was noted in 2 patients under 10 years of age (Cases 12 and 18) and in 7 others under 20 years (Cases 2, 17, 38, 54, 61, 69 and 78), but occurred most commonly in the age group 29–59. Very

TABLE I.—*Summary of the Eighty-five Cases of Post Nasal Space Malignancy Symptoms, Radiological Findings*

*Tribe*

Ky—Kikuyu
Em—Embu
Mu—Meru
Ka—Kamba
Ly—Luyia
Ki—Kisii
Lu—Luo
Ma—Masai
Na—Nandi
Kp—Kipsigis
El—Elgeyo
Tu—Tugen
Te—Teso
In—Indian

*Histology*

Dif.C.—Differentiated Epidermoid Carcinoma.
Ado.C.—Adeno-carcinoma.
Ana.C.—Anaplastic (undifferentiated) Epidermoid carcinoma.
Unc.C.—Unclassified Carcinoma.
Ret.S.—Reticulum Cell Sarcoma.
Lym.S.—Lymphosarcoma.
M.MST.—Malignant Mixed Salivary Tumour.

| | | | | | Ear | | | | Nose | | Eye | | | | Mouth and Pharynx | | | | | | |
|---|---|---|---|---|---|---|---|---|---|---|---|---|---|---|---|---|---|---|---|---|---|
| No. | Sex | Age | Tribe | Duration of symptoms in months | Tinnitus | Deafness | Discharge | Pain | Obstruction | Epistaxis | Blindness | Proptosis | Ptosis | Pain | Dysphagia | Trismus | Ptyalism | Pain | Voice changes | Cough | Dyspnoea |
| 1 | M. | 65 | Ki | 12 | | | | | | | | | | | + | | | | | + | |
| 2 | M. | 16 | Tu | 12 | | | | | + | + | | | + | | | + | | | | | |
| 3 | F. | 30 | Ky | 24 | | | | + | | + | | | | | | + | | | | | |
| 4 | M. | A | Ky | 4 | | | + | + + | | | | | | | | | | | | | |
| 5 | M. | A | Mu | 8 | | | | | + | | + | + | | | + | | | | | | |
| 6 | M. | 28 | Ky | 9 | + | + | | | | + | | | | | | | | | | | |
| 7 | M. | A | Ly | 4 | | + | | | + | | | | | | | | | | | | |
| 8 | M. | A | Ky | 5 | | | | | | | | | | | + | + | | | | + | |
| 9 | M. | A | Kp | 8 | | | | | | | | | | | | | | | | | |
| 10 | M. | A | Ky | 5 | | + | | | + | | | | | | | | | | | | |
| 11 | M. | A | Ky | 12 | + | | | | | + | | | | | | | | | + | | |
| 12 | M. | 7 | Kp | 3 | | | | | + | | | | | | + | | | | | | |
| 13 | M. | 40 | Ka | 8 | | | | | | | | | | | | | | | | | |
| 14 | M. | A | Lu | 2½ | | | | | | | | | | | | | + | | | + | |
| 15 | M. | A | Ky | 2 | | | | | | | | | | | | | | | | | + |
| 16 | M. | 45 | Ly | 24 | | | | | | | | | | | | | | | | | |
| 17 | M. | 15 | Ky | 12 | | | | + + | + | | + | + | + | | | + + | | + + | + | | |
| 18 | F. | 9 | Lu | 3 | | | | | | | | | | | | | | | | | |
| 19 | F. | A | Na | 6 | | | | | | + | | | | | | | | | | | |
| 20 | M. | 50 | Ky | 6 | | | | + | | + | | | | | | | | | | | |
| 21 | M. | A | Ka | 2 | | + | | | | | + | | | + | | | | | | | |
| 22 | M. | 30 | Ma | 2 | | + | | | + | | + + Bilat. | | + | | | | | | | | |

*Admitted to this Series—Showing Personal Details, Duration, Signs and and Histological Diagnosis*

*Signs and Symptoms*

+ —Present.
+ + —Moderately severe.
+ + + —Very severe.

*Radiological Erosion*

+ —Erosion proven.
Nil—No erosion seen.
Blank—Inadequate X-rays for full study.

*P.N.S. Tumour*

+ —Tumour Present.
Nil—No tumour palpable under anaesthesia.
(see also "Remarks" column).

| Neck | | | | | | | | | | | |
|---|---|---|---|---|---|---|---|---|---|---|---|
| Glands | Pain | Headache | Face pain | Fever | Weight loss | Cranial nerves involved | P.N.S. Tumour | Radiological Erosion Base of skull | P.N.S. Histology | Remarks | Distant metatasis |
| + + + | + + | | | | | | Nil | | Ana.C. | Nil felt in P.N.S. | |
| Nil | | + + | + + | | | 5, 6, 7, 8 | + | + | Ana.C. | Small P.N.S. tumour. | |
| + + + | + + | + + | | | | 5 | + | + | Unc.C. | Small P.N.S. tumour. | |
| + + | | | | | | | + | + | Ana.C. | Smooth infiltrating tumour in P.N.S. | |
| + + | | + + | | | | 2, 3, 4, 5, 6 | + | + | Ana.C. | Large fungating tumour in P.N.S. | |
| Nil | | + + | | | | 8, 9, 10, 11, 12 | + | | Dif.C. | Fits. At P.M. middle fossa erosion. | |
| + + + | | + | | | | | + | Nil | Ana.C. | Very vascular large tumour in P.N.S. | |
| + + + | + + | + + | | + | + | | + | | — | Only neck glands biopsied. Ana.C. | |
| + + + | | + + | | | | | Nil | | — | Only neck glands biopsied. Ana.C. | |
| + + + | | + | | | | | + | + | Ana.C. | Hard tumour in P.N.S. | |
| + + | | + + | | | | Partial 5 | + | | | Large tumour in P.N.S. | |
| + + + | | | | | + | | + | | Ana.C. | Large tumour in P.N.S. | |
| + + + | + | | | | + | | + | | Ana.C. | P.N.S. tumour palpated. | |
| + + + | + + | + | | | + | 12 | + | | Unc.C. | Ulcerating tumour in P.N.S. | |
| + + + | | | | | | | + | | — | Tracheostomy on admission. Died next day. Very large P.N.S. tumour | |
| + + + | | | | | | | + | | M.MST | Tumour palpated in P.N.S. | |
| + + | | + + | | | | 2, 3, 4, 5, 6, 7, 8, 9, 10 | + | + | Ana.C. | Ulcerating tumour in P.N.S. | |
| + + + | + + | | | | | | Nil | Nil | Ret.S. | Nil felt in P.N.S. | |
| + + + | + + | | | | + | | + | Nil | Ana.C. | Large vascular tumour in P.N.S. | |
| + + + | + + | + | | | | 6 | Nil | + | Dif.C. | Nil felt in P.N.S. | |
| + + + | + + | + + | | | | 2, 6, 12 | + | + | — | Large ulcerating tumour in P.N.S. Only neck glands biopsied. Ana.C. | Rt. lung |
| Nil | | + + | | | | R/2, 3, 4, 5, 6, 7, 12 L/2, 3, 4, 6 | + | + | Ana.C. | Ulcerating tumour extensively eroding base at P.M. | |

TABLE I (*continued*)

| | | | | | Ear | | | | Nose | | Eye | | | | Mouth and Pharynx | | | | | | |
|---|---|---|---|---|---|---|---|---|---|---|---|---|---|---|---|---|---|---|---|---|---|
| No. | Sex | Age | Tribe | Duration of symptoms in months | Tinnitus | Deafness | Discharge | Pain | Obstruction | Epistaxis | Blindness | Proptosis | Ptosis | Pain | Dysphagia | Trismus | Ptyalism | Pain | Voice changes | Cough | Dyspnoea |
| 23 | M. | A | Ly | 2 | | | | | | + | | | | | | | | | | | |
| 24 | M. | 36 | Te | 24+ | | | | | | | | | | | + | | | | + | | |
| 25 | M. | 30 | Ky | 16 | | | | | | + | | | | | + | | | | | | |
| 26 | M. | A | Ka | 4 | | + | | + | | | | | | | + | | | | | + | |
| 27 | M. | 50 | Ma | ?6 | | | | | | +++ | | | | | | | | | | | |
| 28 | F. | 25 | Na | 6 | | + | | | | | | | | | | | | | | | |
| 29 | F. | 20 | Ka | 1 | | | | | | | | | | | | + | | ++ | | | |
| 30 | M. | A | Ly | 6 | | + | | | + | | | | | | | | | | | | |
| 31 | F. | 20 | Ki | 9 | | + | | + | | | | | + | + | + | + | | | | | |
| 32 | F. | A | Ky | 12 | | | | | | | | | | | | | | | | | |
| 33 | F. | 40 | Ky | 12 | + | + | | | | | | | | | + | | | | | | |
| 34 | F. | 50 | Ma | 1 | | + | | | | | | | | | | | | | | | |
| 35 | M. | 30 | Lu | 3 | | | | | | | | | | | | | | | | | |
| 36 | M. | 48 | Lu | 3 | | | + | | + | + | + | | | | | | | | | | |
| 37 | F. | 40 | Ky | 6 | | | | | | | | | | | | | | | | | |
| 38 | F. | 18 | Mu | 4 | | | | + | | + | | | | | | | | | | | |
| 39 | M. | 70 | Ka | 3 | | | | | | | | | | | | + | | | | | |
| 40 | M. | 75 | Ka | 12 | | + | + | + | | | | | | | | | | | + | | |
| 41 | M. | A | Ky | 1 | | | | | | | | + | | | | | | | | | |
| 42 | F. | 48 | Kp | 9 | | | | | | +++ | | | | | | + | | | | | |
| 43 | F. | 60 | Ly | 1 | | | | | | | | | | | + | | | | + | | |
| 44 | F. | 50 | Ky | 4 | | + | | | + | | | | | | + | + | | | + | + | |
| 45 | M. | 50 | Ky | 18 | | | | | | | | | + | | | | | | + | | |
| 46 | M. | 40 | Ky | 3 | | | | + | | | | | | | + | | | + | | | |
| 47 | M. | 50 | Em | 4 | | + | | | + | | | | | | | + | | | | | |
| 48 | M. | A | Ky | 2 | | | | | | | | | | | | | | | | | |
| 49 | M. | 50 | Ky | 2 | | | | | | | | | | | | | | | | + | |
| 50 | F. | 30 | Kp | 3 | | | | | | | | | + | | + | + | | | | | + |

| Neck | | Headache | Face pain | Fever | Weight loss | Cranial nerves involved | P.N.S. Tumour | Radiological Erosion Base of skull | P.N.S. Histology | Remarks | Distant metastasis |
|---|---|---|---|---|---|---|---|---|---|---|---|
| Glands | Pain | | | | | | | | | | |
| + + + | + + | | | | | | + | | Ana.C. | Large ulcerating vascular tumour in P.N.S. | |
| + + + | | + | | | | | + | | Unc.C. | Large ulcerating tumour in P.N.S. | |
| + + + | | | | | | | + | | — | Large ulcerating P.N.S. tumour. Only neck gland biopsy. Ana.C. | |
| + + | + + | | | | | | + | | | Large tumour in P.N.S. | |
| + | | | | | + | | + | | — | Large vascular friable tumour in P.N.S. Died on admission | |
| + + + | + + | + | | | | | + | | Ana.C. | Large ulcerating growth. | |
| + | | + | | | | 4, 5, 6 | + | + | Ana.C. | Tumour palpated in P.N.S. | |
| + + + | + + | + | | | | | Nil | + | Unc.C. | Nil felt in P.N.S. | |
| + + + | + + | + | | | | (Brachial plexus lesion) | + | | Dig.C. | Ulcerating tumour | T10 vertebrae |
| + + + | + + | | | + | | | + | + | Ana.C. | Large P.N.S. tumour | |
| + + + | + + | | | | | | Nil | | Unc.C. | Nil felt in P.N.S. | |
| + + + | + + | | | | | | + | | Ana.C. | Large tumour in P.N.S. | |
| + + + | + + | | | | | | + | + | Ana.C. | Nodular tumour in P.N.S. | Lower lumbar spine |
| + + + | | | | | | 2, 6 | + | + | — | Large hard tumour in P.N.S. Only neck gland biopsy. Ana.C. | |
| + + + | + + | + + | | | | | + | + | Ana.C. | Large tumour in P.N.S. | |
| + + | + | | | | | | + | Nil | Ana.C. | Large tumour in P.N.S. | |
| + + + | + + | + + | | | | 12 (Brachial plexus lesion) | + | + | Lym.S. | Large tumour in P.N.S. | |
| Nil | | | | | | 5, 6, 7 | + | + | Ado.C. | Large tumour in P.N.S. and in external auditory meatus | |
| + + + | + + | | | | | 2, 3, 4, 5, 6 | + | | Ana.C. | Large ulcerating tumour | |
| + | + | | | | | | + | Nil | Dif.C. | Small P.N.S. tumour | |
| + + | | + + | | | | | + | | Ret.S. | Large friable vascular tumour in P.N.S. | |
| + + + | + + | | | | | | + | + | Ret.S. | Large tumour in P.N.S. | Both lungs |
| + + + | | | | | | 12 | + | | Ana.C. | Small tumour in P.N.S. | |
| + + + | + + | | | | + | | + | + | Ado.C. | Small tumour in P.N.S. | |
| + + + | + + | + + | | | | 9 | + | + | — | Small tumour in P.N.S. Neck gland biopsy only. Ana.C. | |
| + + + | | | | | | | + | | Dif.C. | Small ulcerating tumour in P.N.S. | |
| + + + | | | | | | | + | | — | Small tumour in P.N.S. Neck gland biopsy only. Ana.C. | Liver. Spleen Mediastinum |
| + + + | | | | | | 3, 4, 6 | + | + | Unc.C. | Large P.N.S. tumour | Carcinomatosis (subcutaneous) |

TABLE I (*continued*)

| No. | Sex | Age | Tribe | Duration of symptoms in months | Ear | | | | Nose | | Eye | | | | Mouth and Pharynx | | | | | | |
|---|---|---|---|---|---|---|---|---|---|---|---|---|---|---|---|---|---|---|---|---|---|
| | | | | | Tinnitus | Deafness | Discharge | Pain | Obstruction | Epistaxis | Blindness | Proptosis | Ptosis | Pain | Dysphagia | Trismus | Ptyalism | Pain | Voice changes | Cough | Dyspnoea |
| 51 | M. | 55 | Mu | 6 | | + | | | | + | | | + | | + | | | | | | + |
| 52 | M. | 55 | Na | 8 | | | | | | + | | | | | | | | | | | |
| 53 | M. | 30 | Na | 4 | | + | | | + | | | + | + | | | + | | | | | |
| 54 | M. | 19 | Mu | 27 | | + | | + | + | | | | | | | + | + | | + | | + |
| 55 | M. | 22 | Ly | 1 | | + | | + | + | | | + | | | + | + | + | | | | |
| 56 | F. | 55 | Ky | 3 | | | | | | | | | | | + | | | | + | + | |
| 57 | M. | 50 | Ky | 6 | | + | | | | + | | | | | | | | | | + | |
| 58 | M. | 26 | Ly | 3 | + | + | | | + | | | + | | | | + | | | | | |
| 59 | F. | 35 | Lu | 4 | | | | + | | | | | | | | + | | | | | |
| 60 | M. | 50 | Ky | 7 | | + | | | | + + | | | | | | | | + | | | |
| 61 | M. | 17 | Ky | 4 | | + | | | | | | | | | | | | | | | |
| 62 | M. | 35 | Ki | 12 | | | | | | + | | | | | + | | | + | | | |
| 63 | M. | A | Ky | 30 | | | + | + | | | + | | + | | | | | | | | |
| 64 | M. | 55 | El | 24 | | + | | | + | | | | | | + | | | | | | |
| 65 | M. | A | Em | 30 | | + | | | | | | | | | + | | | | | | |
| 66 | M. | 60 | Na | 12 | | + | | + | | | | | | | + | + | | | | | |
| 67 | M. | 26 | Ly | 3 | | | | | | | | + | | | + | | | | + | | |
| 68 | M. | 34 | Ly | 8 | + | | | | + | + + | | | | | | | | | | | |
| 69 | M. | 14 | Na | 6 | | | | | | | | + | + | | + | + | | | | | |
| 70 | M. | 49 | Ly | 7 | | | | | | | | | | | | | | | | | |
| 71 | M. | 27 | Na | 18 | | | | | + | | | | | | + | | | | + | | |
| 72 | M. | 40 | Ky | 9 | | | | + | + | | | + | + | | | | | | | | |
| 73 | M. | 37 | Ky | 12 | | | | | | | | | | | + | | | | | | |
| 74 | F. | 45 | Ky | 8 | | | | | + | + | | | | | | | | | | | |
| 75 | M. | 50 | Ky | 18 | | + + | | | | | | | | | | | | | | | |
| 76 | M. | 54 | In | 1 | | | | | | | | | | | | | | | | | |
| 77 | M. | 35 | Na | 12 | | | | | | + | | | | | | | | | | | |
| 78 | F. | 15 | Ki | 9 | | | | + | | + | | | | | | | | | | | |
| 79 | M. | 34 | Ky | 9 | | | | + | | | | | | | | | | | | | |
| 80 | F. | 35 | Ky | 2 | | | | | | | | | | | + | | | | | | + |
| 81 | F. | 35 | Ky | 4 | | | | | | | | | | | + | | | + | | | + |
| 82 | M. | 63 | Ly | 3 | | | | | | | + | | | | | | | | + | | + |
| 83 | F. | 23 | Mu | 7 | | | | | + | + | | | | | | | | | | | |
| 84 | M. | 47 | In | 3 | | | | | + | + | | | | | | | | | | | |
| 85 | F. | 50 | Kp | 4 | | | | | + | + | | | | | | | | | | | |

| Neck | | | | | | | | | | | |
|---|---|---|---|---|---|---|---|---|---|---|---|
| Glands | Pain | Headache | Face pain | Fever | Weight loss | Cranial nerves involved | P.N.S. Tumour | Radiological Erosion Base of skull | P.N.S. Histology | Remarks | Distant metastasis |
| +++ | ++ | + | + | | | 5 | + | + | — | Large nodular P.N.S. tumour. Only neck gland biopsy. Ana.C. | |
| +++ | ++ | ++ | | | | | + | | Unc.C. | Small tumour in P.N.S. | |
| +++ | ++ | ++ | | | | 2, 3, 7, 8, 12 | + | + | Ana.C. | Large vascular P.N.S. tumour | |
| +++ | | | | | | 5, 6, 11, 12 | + | + | Ana.C. | Enormous tumour in P.N.S. | |
| +++ | ++ | ++ | | | | 5, 6, 11, 12 | + | + | Ado.C. | Large P.N.S. tumour | |
| +++ | ++ | | | | + | | + | Nil | Lym.S. | Large tumour in P.N.S. | |
| +++ | | ++ | | | | | + | | Ana.C. | Medium size tumour in P.N.S. | |
| +++ | ++ | ++ | | | | | + | + | Ana.C. | Large tumour in P.N.S. | |
| +++ | ++ | ++ | | | | 3, 4, 6 | + | Nil | Unc.C. | Large tumour in P.N.S. | |
| Nil | | | | | | | + | Nil | Unc.C. | Small tumour in P.N.S. | |
| +++ | ++ | ++ | | | | 7, 8, 11 | + | | Ana.C. | Medium size tumour in P.N.S. | |
| ++ | + | | | | | | + | | Ret.S. | Very large tumour | Groin gland |
| + | | ++ | | | | 2, 3, 4, 5, 6, 7, 8, 9, 10, 11, 12 | Nil | | Ana.C. | Nil felt in P.N.S. | |
| +++ | | | | | | | + | Nil | Ana.C. | Large P.N.S. tumour | |
| +++ | | | | | | 9, 10, 11, 12 | + | + | Ana.C. | Small tumour in P.N.S. | |
| +++ | ++ | + | | | | 3, 6, 7, 8, 9, 12 | + | | Ana.C. | Large tumour | |
| + | | ++ | | | | 9, 10, 11, 12 (+ brachial plexus) | + | | Dif.C. | Large ulcerating tumour | |
| Nil | | ++ | | | | | + | | Unc.C. | Large tumour in P.N.S. | |
| +++ | ++ | | + | | | 3, 4, 5, 6 | + | + | Ana.C. | Large tumour in P.N.S. | Tumour eroding through to temporal lobe |
| +++ | ++ | | | | | | + | Nil | Ado.C. | Large friable tumour | |
| + | + | + | | | | 9, 10 | + | + | Ana.C. | Hard smooth tumour in P.N.S. | |
| +++ | ++ | | | | | | + | + | Dif.C. | Large tumour in P.N.S. | Liver and rib |
| +++ | | ++ | | | | | + | + | Dif.C. | Large tumour in P.N.S. | |
| +++ | | | | | | | + | | Ana.C. | Large tumour in P.N.S. | |
| +++ | | | | | | | + | | Ana.C. | Small P.N.S. tumour | |
| +++ | | | | | | | + | | Ana.C. | Small tumour in P.N.S. | |
| +++ | ++ | | | | | | + | | Ado.C. | Large tumour in P.N.S. | |
| +++ | ++ | ++ | + | | | | + | + | Dif.C. | Small tumour in P.N.S. | |
| +++ | ++ | ++ | | | | | + | | — | Large ulcerating tumour in P.N.S. Neck gland only. Ret.S. | Liver and groin |
| +++ | ++ | | | | | | + | Nil | — | Large P.N.S. tumour. Neck glands only. Lym.S. | |
| + | + | | | | | | + | | Ret.S. | Large P.N.S. tumour | |
| +++ | | | | | | | + | | Ret.S. | Large P.N.S. tumour | |
| Nil | | | | | | 2, 3, 4, 5, 6 | + | | Ana.C. | Friable vascular large tumour | |
| Nil | | | | | | | + | Nil | Dif.C. | Large friable vascular tumour | |
| Nil | | | + | | | | + | | Dif.C. | Medium size tumour in P.N.S. | |

Fig. 1.—Ethnographic Map of Kenya.

few African patients know their age accurately and in many cases it is impossible to make a reliable estimate, in this series these are classified as " adult ".

*Ethnological Classification*

Apart from the immigrant races (European and Indian) there are four main ethnic groups living in Kenya, and the areas of the country which they occupy are shown in Fig. 1 and the geographical distribution of cases is shown in Fig. 2. No case has been recorded in the Hamitic group (the African branch of the Caucasian family). The incidence in the three other groups, Bantu, Nilotic and Nilo-Hamitic, is noted in Table III.

FIG. 2.—Geographical distribution of cases.

Key
● = Male
× = Female
- - - - = Relevant tribal boundaries (see also Fig. 1)

Two cases were Indians (82 and 90) and no case presented from the European community. The population of Kenya is divided as follows, according to the Kenya Census Report 1963.

*Immigrant races*

| | | | |
|---|---|---|---|
| *European* | | | 55,822 |
| *Asian* | | | |
| Indian, Pakistani, Goan | | 176,879 | |
| Arabs | | 34,263 | 215,051 |
| Others | | 3,909 | |

*African natives*

| | | *Male* | *Female* | *Total* |
|---|---|---|---|---|
| *Bantu* | Central | 1,550,049 | 1,613,657 | |
| | Nyanza | 822,311 | 847,350 | 5,362,045 |
| | Coastal | 254,957 | 273,721 | |
| *Nilotic* | | 559,093 | 585,017 | 1,144,110 |
| *Nilo-Hamitic* | | 671,588 | 683,538 | 1,355,126 |
| *Hamitic* | | | | 372,400 |

It is of interest to note that, though females slightly outnumber males in the three ethnic groups, the ratio in hospital patients with nasopharyngeal cancer was 5 males to 2 females. No case has been noted in the Coastal Bantu who live under different environmental conditions from the Central (Kikuyu, Embu, Meru and Kamba) and Nyanza (Luyia and Kisii) Bantu (Fig. 1, 2 and 12).

TABLE III.—*Tribal Incidence*

(actual and expected incidence by Tribe is further developed by Linsell, 1964)

| Ethnic Group | Tribe | No. | Per cent | No. | Per cent |
|---|---|---|---|---|---|
| Bantu | Kikuyu | 31 | 36·4 | | |
| | Embu | 2 | 2·4 | | |
| | Meru | 5 | 5·9 | | |
| | Kamba | 6 | 7·0 | 59 | 69·3 |
| | Luyia | 11 | 12·9 | | |
| | Kisii | 4 | 4·7 | | |
| Nilotic | Luo | | | 5 | 5·9 |
| Nilo-Hamitic | Masai | 3 | 3·5 | | |
| | Nandi | 8 | 9·4 | | |
| | Kipsigis | 5 | 5·9 | 19 | 22·4 |
| | Elgeyo | 1 | 1·2 | | |
| | Tugen | 1 | 1·2 | | |
| | Teso | 1 | 1·2 | | |
| Indian | | | | 2 | 2·4 |
| Total | | | | 85 | 100·0 |

*Clinical Presentation*

Table IV shows the relative incidence of the presenting symptoms.

TABLE IV.—*Presenting Symptoms*

| | | | No. | Per cent |
|---|---|---|---|---|
| Neck : | Painless glands | | 13 | 15·6 |
| | Painful glands | | 37 | 43·5 |
| Other Pain : | Head | | 24 | 28·2 |
| | Face | | 6 | 7·0 |
| | Ear | | 4 | 4·7 |
| Others : | Nose : | Epistaxis | 9 | 10·6 |
| | | Obstruction | 3 | 3·5 |
| | Eye : | Blindness | 3 | 3·5 |
| | Mouth : | Trismus | 4 | 4·7 |
| | | Dysphagia | 13 | 15·6 |
| | All Others | | 11 | 12·9 |

The clinical pattern of the disease in Kenya is similar to that noted by Digby *et al.* (1941) and Liang Po-Chiang *et al.* (1962) in the Chinese, but in contrast to Lederman's (1961) study of 218 English and Maltese patients, headache occurred more frequently in the African as a presenting symptom.

It was possible to subdivide these 85 cases into four clinical groups (see Table V) :

TABLE V.—*Clinical Type*

| Type | No. | Per cent | Number with distant metastases |
|---|---|---|---|
| 1. Signs of local effects only i.e. in immediate vicinity of P.N.S. | 5 | 5·9 | – |
| 2. Glandular enlargement without cranial nerve lesions | 49 | 57·8 | 7 |
| 3. Glands and cranial nerve lesions : | | | |
| (a) Glands predominating | 15 | 17·6 | 1 |
| (b) Nerve lesions predominating | 6 | 7·0 | – |
| (c) Both glands and nerve lesions marked | 6 | 7·0 | 2 |
| 4. Nerve lesions with no gland involvement | 4 | 4·7 | – |

*Note :*

1. Distant metastases do not seem to occur where nerve lesions are the predominant feature.
2. No case in Group 1 had either signs of spread beyond the immediate vicinity of the P.N.S. or of bony erosion, *but* 25 cases in Group 2 (together with 1 each in Groups 3a, 3c and 4) had no signs of direct spread beyond the immediate vicinity of the P.N.S.—this included 2 cases with distant metastases.

(1) Patients presenting with symptoms and signs referable to local disease.

(2) Patients presenting with cervical gland involvement (Fig. 3 and 4).

(3) Patients presenting with enlarged cervical glands and cranial nerve lesions (Fig. 5 and 6).

(*a*) Gland involvement predominating.

(*b*) Cranial nerve lesion predominating.

(*c*) With marked involvement of both cervical glands and cranial nerves.

(4) Cranial nerve lesions without cervical glands (Fig. 7 and 8).

It will be noted that the commonest clinical presentation (Table V, type 2) was that of massive cervical glandular involvement without a cranial nerve lesion (57·8 per cent). Lederman (1961) found evidence of cranial nerve involvement in 26·6 per cent of his patients but in only 3·2 per cent were these the presenting symptom. In this series 35·5 per cent of the patients were found to have involvement of one or more cranial nerves, which in 3·5 per cent (all due to blindness) caused the patient to seek treatment. The incidence of individual cranial nerve involvement in this series is shown in Table VI and Lederman's (1961) findings are included for comparison. It is of interest to note that one case (No. 67) presented as Garcin's Syndrome and that the 7th and 8th cranial nerves were involved much more frequently in the African patients, indicating an advanced stage of the disease when the patient first presented.

### *Radiological Findings*

In 45 of these patients adequate X-rays of the base of the skull, or the report of a full examination, were available for review, and the incidence of a radio-

TABLE VI.—*Cranial Nerve Involvement*

Thirty-one cases (35·5 per cent of total) one of which had bilateral nerve lesions. These figures are compared with those in the series described by Lederman (1961) in which 26·6 per cent had nerve involvement.

| Cranial nerve involved | No. | Kenya per cent (of total 85) | Lederman, 1961, English and Maltese per cent (of total 218) |
|---|---|---|---|
| I | — | — | 0·5 |
| II | 8 | 9·4 | 4·0 |
| III | 10 | 11·8 | 9·0 |
| IV | 9 | 10·6 | 8·0 |
| V | 13 | 15·6 | 16·0 |
| VI | 17 | 20·0 | 14·0 |
| VII | 8 | 9·4 | 3·0 |
| VIII | 7 | 8·2 | 1·0 |
| IX | 8 | 9·4 | 6·0 |
| X | 6 | 7·0 | 9·0 |
| XI | 7 | 8·2 | 8·0 |
| XII | 13 | 15·6 | 6·0 |

logically demonstrated base of skull erosion as related to the clinical type of the disease is shown in Table VII.

TABLE VII.—*Radiological Evidence of Erosion Related to Clinical Type 45 Cases in Which Full X-ray Studies Were Available*

| Type (as in Table V) | 1 | 2 | 3a | 3b | 3c | 4 |
|---|---|---|---|---|---|---|
| Radiological erosion of bone | 0 | 12 | 7 | 3 | 7 | 3 |
| No erosion seen on X-ray | 3 | 9 | 1 | 0 | 0 | 0 |

Of the 24 patients who showed no evidence of cranial nerve involvement (Types 1 and 2), only 9 had radiological evidence of erosion of the base of the skull, in contrast to the 21 patients presenting with a cranial nerve lesion (Types 3 and 4), 20 of whom had base of skull erosion. Table VIII shows how bone erosion was related to the incidence of individual cranial nerve lesions, the 5th and 6th cranial nerves being the most commonly involved.

As might be expected, cranial nerve lesions occurred most frequently in patients with radiological evidence of bone erosion, and only one patient (No. 63)

EXPLANATION OF PLATES

FIG. 3.—Case 10. Adult male Kikuyu (Central Bantu). Bilateral cervical gland masses secondary to anaplastic carcinoma of nasopharynx. No cranial nerve involvement.

FIG. 4.—Case 28. 25 year old female Nandi (Nilo-Hamitic). Right cervical gland mass. Anaplastic carcinoma nasopharynx. No cranial nerve involvement.

FIG. 5.—Case 5. Adult male Meru (Central Bantu). Large left cervical gland mass. Right 2, 3, 4, 6 and left 5 cranial nerve paralysis. Anaplastic carcinoma nasopharynx.

FIG. 6.—Case 41. Adult male Kipsigis (Nilo-Hamitic). Bilateral cervical glands. Left 2, 3, 4, 5 and 6 cranial nerve paralysis. Anaplastic carcinoma nasopharynx.

FIG. 7.—Case 6. 28 year old Kikuyu (Central Bantu). Left 8, 9, 10, 11, 12 cranial nerve paralysis. Differentiated epidermoid tumour in nasopharynx. No cervical glands.

FIG. 8. Case 83. 23 year old female Meru (Central Bantu). Right 2, 3, 4, 5, 6 cranial nerve paralysis. Anaplastic carcinoma nasopharynx. No cervical glands.

FIG. 9.—A typical African hut in Central Province. Cooking fire smoke is seen escaping through the grass thatch roof. These huts are constructed without a chimney.

3

4

5

6

7

8

9

TABLE VIII.—*Radiological Evidence of Bony Erosion Related to Individual Cranial Nerve Lesions*

| | II | III | IV | V | VI | VII | VIII | IX | X | XI | XII | Total number with nerve lesions | Total number with no nerve lesion |
|---|---|---|---|---|---|---|---|---|---|---|---|---|---|
| Proved erosion on X-ray | 7 | 7 | 7 | 11 | 14 | 5 | 3 | 4 | 3 | 3 | 6 | 20 | 12 |
| No erosion on X-ray | – | 1 | 1 | — | 1 | – | – | – | – | – | – | 1 | 12 |
| Inadequate X-ray studies | 1 | 2 | 1 | 2 | 2 | 3 | 4 | 4 | 3 | 4 | 6 | 8 | 32 |

had a cranial nerve lesion without evidence of bone erosion : in this instance involvement of the 3rd, 4th and 6th cranial nerves was probably due to direct extension of the disease through the inferior orbital fissure. The radiological aspects of this disease in the Kenya African are described in detail by Whittaker (1964).

TABLE IX.—*Histological Type*

(Seventy-one cases have P.N.S. biopsy, 10 cases have only neck gland biopsy in conjunction with clinical findings, and in 4 there is only a clinical diagnosis)

| Histological classification | No. | Per cent |
|---|---|---|
| Differentiated epidermoid carcinoma | 11 | 12·9 |
| Adenocarcinoma (including 1 malignant mixed salivary tumour) | 6 | 7·0 |
| Anaplastic (undifferentiated) epidermoid carcinoma | 44 | 51·9 |
| Unclassified carcinoma | 10 | 11·8 |
| Reticulum cell sarcoma | 7 | 8·2 |
| Lymphosarcoma | 3 | 3·5 |
| Clinical diagnosis only (2 died—no biopsy and 2 biopsy specimens unsatisfactory) | 4 | 4·7 |

### *Histology*

The histological classification of the cases reported in this study is described by Linsell (1964) and the incidence of different histological types is noted in Table IX. Anaplastic (undifferentiated) epidermoid carcinoma was the commonest type (51·9 per cent).

TABLE X.—*Presenting Symptoms Related to Histological Diagnosis*

| | Neck | | Pain | | | | | | | | |
|---|---|---|---|---|---|---|---|---|---|---|---|
| | Painless glands | Painful glands | Head | Face | Ear | Epistaxis | Nasal obstruction | Blindness | Trismus | Dysphagia | Others |
| Differentiated epidermoid carcinoma | 2 | 4 | 3 | – | 1 | 4 | 2 | – | – | 1 | 1 |
| Adenocarcinoma | 1 | 3 | – | – | – | – | – | – | – | 1 | 1 |
| Anaplastic epidermoid carcinoma | 10 | 17 | 14 | 4 | 3 | 2 | 1 | 3 | 3 | 5 | 5 |
| Unclassified carcinoma | – | 6 | 3 | – | – | 2 | – | – | 1 | 1 | – |
| Reticulum cell sarcoma | – | 3 | 2 | 2 | – | – | – | – | – | 3 | 2 |
| Lymphosarcoma | – | 3 | 1 | – | – | – | – | – | – | 1 | 1 |
| No histology | – | 1 | 1 | – | – | 1 | – | – | – | 1 | 1 |

TABLE XI.—*Histology Related to Clinical Type*

Figures in brackets show cases with distant metastases. Of the 4 cases without histological diagnosis, 3 were on Type 2 and 1 of Type 3a.

| | Differentiated epidermoid carcinoma | Adeno-carcinoma | Anaplastic (undifferentiated) epidermoid carcinoma | Unclassified carcinoma | Reticulum cell sarcoma | Lympho-sarcoma |
|---|---|---|---|---|---|---|
| Type 1 | 2 | 0 | 1 | 2 | 0 | 0 |
| Type 2 | 5 (2) | 4 | 24 (2) | 4 | 7 (3) | 2 |
| Type 3a | 1 | 0 | 8 | 4 (1) | 0 | 1 |
| Type 3b | 1 | 0 | 5 | 0 | 0 | 0 |
| Type 3c | 1 | 1 | 4 (2) | 0 | 0 | 0 |
| Type 4 | 1 | 1 | 2 | 0 | 0 | 0 |
| Total | 11 | 6 | 44 | 10 | 7 | 3 |

TABLE XII.—*Duration of Symptoms in Relation to the Histology of the Tumour*

The history is likely to be very inaccurate, and few patients know exactly how long they have had symptoms.

| | Duration (in months) | | | | |
|---|---|---|---|---|---|
| | 0–3 | 4–6 | 7–9 | 10–12 | 13 and over |
| Differentiated epidermoid carcinoma . | 3 | 2 | 5 | 1 | — |
| Adenocarcinoma . . . | 2 | — | 1 | 2 | 1 |
| Anaplastic epidermoid carcinoma . | 12 | 14 | 5 | 5 | 8 |
| Unclassified carcinoma . . | 2 | 2 | 3 | 1 | 2 |
| Reticulum cell sarcoma . . | 3 | 2 | 1 | 1 | — |
| Lymphosarcoma . . . | 3 | — | — | — | — |
| Unknown histology . . | 1 | 2 | — | 1 | — |
| Total . . . . . . | 26 | 22 | 15 | 11 | 11 |

The relationship between the histological diagnosis and the presenting symptoms, the clinical type and the duration of symptoms are outlined in Table X, XI and XIII. As will be seen from Tables X and XI, enlarged cervical glands occurred with all histological types and symptoms relating to these large secondary gland masses formed the commonest presenting symptoms. Symptoms due to cranial nerve involvement occurred in approximately 50 per cent of cases classified as differentiated epidermoid, adenocarcinoma, and anaplastic epidermoid carcinoma; this was in contrast to the low incidence of cranial nerve involvement by tumours classified as "unclassified" carcinoma, reticulum cell sarcoma and lymphosarcoma (Table XI). Seventy-four cases (87 per cent) had a history of disease of 12 months or less and the duration of symptoms before the patient presented for treatment did not appear to be related to the histology, except for the lymphosarcomas (Table XII) all 3 of which presented within 3 months of noting symptoms.

### *Nasopharyngeal Carcinoma—Possible Aetiological Factors*

(a) *Environmental*

Steiner (1954) and Pang (1959) have suggested that this disease in the Chinese is due to a genetic or racial susceptibility but Martin and Quan (1951) quote Dobson's view that smoke from kerosene lamps, candles, etc. in poorly ventilated, overcrowded, Chinese houses may be a causative factor. In this series the disease did not affect members of the Bantu ethnic group living at the Coast, whereas some members of this group living under different environmental conditions developed the disease. The geographical distribution of cases is shown in Fig. 1 ; comparison with Fig. 10 and 11 shows that the disease occurs in areas above 2000 feet in altitude, with an annual rainfall of over 20 inches. Fig. 10 shows

FIG. 10.—Rainfall.

that these are also the areas of greatest population density. The African population in these areas live in small ill-ventilated huts constructed of mud and wattle with a grass thatched roof without a chimney. The disease is not evident in the Coast and Northern Province which are dry and warm and cooking is generally undertaken outside the hut, in contrast to the colder and higher areas (where this disease occurs) where there is a cooking fire in the hut most of the day. The only means of escape for cooking fire smoke is through the grass roof (Fig. 9).

Local wood is used for cooking fires, and Fig. 12 shows the distribution of trees which provide firewood in Kenya.

It will be noted from Fig. 2 and 12 that in those areas in Kenya where the disease occurs exotic trees (eucalyptus and wattle) and the indigenous acacias,

**Fig. 11.—Altitude.**

FIG. 12.—Kenya, Wood Fuel Distribution. Areas are approximate and show principal fuels only ; no one type is used exclusively. (Drawn from a map and information provided by W. E. Dyson of the East African Agriculture and Forestry Research Organisation.)

Scrub, semi-desert and desert. Many species but sparse in wide areas.
Heavy indigenous afforestation. Many types of fuel.
Grassy savannah. Fuel mainly indigenous acacia.
Exotic fuels. Mainly wattle—Acacia Mearnsii de Wild.
Exotic fuels. Mainly gums—Eucalyptus Saligna and Eucalyptus Citriodera. } Indigenous fuels cut out due to cultivation.

which belong to the same Mimosa family as wattle, are used to provide firewood. Specimens of soot taken from the interior of huts of patients with nasopharyngeal carcinoma have been examined by Hoffmann and Wynder (Wynder, 1963) and were found to contain significant amounts of benzopyrene, benzanthracene and other polynuclear aromatic hydrocarbons. More complete details of this work will be reported subsequently. Possibly the inhalation of cooking fire smoke for several hours a day over a period of years, where eucalyptus, wattle and acacias are used as fuel, has some bearing on the incidence of the disease in Kenya.

(b) *Hormonal*

Marsden (1958) believes that there must be more than an external carcinogen to explain the racial predilection shown by this cancer, and this possibly may be hormonic. In this series of Kenya African patients the sex incidence was similar to that reported by Marsden (1958) and Muir (1962) in the Chinese and Malaysian population in Malaya and Singapore ; males were affected approximately twice as frequently as females. Allbrook (1956) has found that the average size of the adrenal cortex of East African males is less than that of White Americans. Politzer and Tucker (1958) have reported that the excretion of ketogenic steroids was significantly lower in Bantu men, and Ch'en P'ei-en (1956) reported that the excretion of 17-ketosteroids was lower in Chinese males and females than in whites. It may be significant that the highest incidence of this disease occurs in populations with low adrenocortical activity.

SUMMARY

1. Malignant disease of the nasopharynx is the commonest head and neck tumour in the Kenya African.

2. The clinical presentation of the disease in 85 patients is described and is similar to that seen in South and East Asia.

3. Anaplastic carcinoma was the commonest histological type. The clinical presentation bore no relationship to the histological diagnosis.

4. Some factors relating to the social and environmental background of these cases, which may have aetiological significance, are described.

We wish to acknowledge with gratitude the assistance we have received from the following : Dr. W. U. Gardner and the Trustees of the Anna Fuller Fund for their generous financial help in this study ; Dr. E. L. Wynder and Dr. D. Hoffmann of the Sloan Kettering Institute, New York, who have examined soot samples for carcinogens ; Mr. B. Shaw and Mrs. E. Willcox of the Health Education Unit, Ministry of Health, Kenya, who have prepared the maps and figures ; Dr. V. G. C. Blackler of the Statistics Department of the Kenya Treasury, for details of the statistics of the ethnic groups in Kenya ; Mr. W. G. Dyson of the East African Agriculture and Forestry Organisation, for providing information on the wood fuel distribution in Kenya.

### REFERENCES

ALLBROOK, D.—(1956) *Lancet*, ii, 606.
CH'EN P'EI-EN—(1956) *Chin. med. J.*, **74**, 424.

Clifford, P.—(1961) *J. Laryng.*, **75**, 707.—(1962) VIII International Cancer Congress. *Acta Un. int. Cancr.* (1964) **20**, (In press).
Digby, K. H., Fook, W. L. and Che, Y. T.—(1941) *Brit. J. Surg.*, **28**, 517.
Lederman, M.—(1961) ' Cancer of the Nasopharynx '. Springfield, Illinois, U.S.A. (Thomas).
Liang Po-Chiang, Chen Gian-Ching, Tsuh Ja-Tsen, Hwu Yuang-Fan, Tsu Shaw-Man and Tsung Yung-Sun (1962) ' Selected papers on Cancer Research '. Shanghai, China (Shanghai Scientific and Technical Publishers).
Linsell, C. A.—(1964) *Brit. J. Cancer*, **18**, 49.
Marsden, A. T. H.—(1958) *Ibid.*, **12**, 161.
Martin, H. and Quan, F.—(1951) *Ann. Otol. etc. St. Louis*, **60**, 168.
Muir, C. S.—(1962) *Brit. J. Cancer*, **16**, 583.
Pang, L. Q.—(1959) *Ann. Otol., etc., St. Louis*, **68**, 356.
Politzer, W. M. and Tucker, B.—(1958) *Lancet*, ii, 778.
Steiner, P. E.—(1954) ' Cancer : Race and Geography ' Baltimore (Williams and Wilkins).
Whittaker, L. R.—(1964) *Brit. J. Cancer*, **18**, 44.
Wynder, E. L.—(1963). Personal communication, quoting Dietrich Hoffman.